# Defective minor spliceosomes induce SMA-associated phenotypes through sensitive intron-containing neural genes in *Drosophila*

**DOI:** 10.1038/s41467-020-19451-z

**Published:** 2020-11-05

**Authors:** Liang Li, Zhan Ding, Ting-Lin Pang, Bei Zhang, Chen-Hui Li, An-Min Liang, Yu-Ru Wang, Yu Zhou, Yu-Jie Fan, Yong-Zhen Xu

**Affiliations:** 1 Key Laboratory of Insect Developmental and Evolutionary Biology, CAS Center for Excellence in Molecular Plant Science, Chinese Academy of Sciences, Shanghai, 200032 China; 2 University of Chinese Academy of Sciences, Shanghai, 200032 China; 3 State Key Laboratory of Virology, Hubei Key Laboratory of Cell Homeostasis, College of Life Science, Wuhan University, Hubei, 430072 China

**Keywords:** RNA, RNA splicing, Molecular neuroscience

## Abstract

The minor spliceosome is evolutionarily conserved in higher eukaryotes, but its biological significance remains poorly understood. Here, by precise CRISPR/Cas9-mediated disruption of the U12 and U6atac snRNAs, we report that a defective minor spliceosome is responsible for spinal muscular atrophy (SMA) associated phenotypes in *Drosophila*. Using a newly developed bioinformatic approach, we identified a large set of minor spliceosome-sensitive splicing events and demonstrate that three sensitive intron-containing neural genes, *Pcyt2, Zmynd10*, and *Fas3*, directly contribute to disease development as evidenced by the ability of their cDNAs to rescue the SMA-associated phenotypes in muscle development, neuromuscular junctions, and locomotion. Interestingly, many splice sites in sensitive introns are recognizable by both minor and major spliceosomes, suggesting a new mechanism of splicing regulation through competition between minor and major spliceosomes. These findings reveal a vital contribution of the minor spliceosome to SMA and to regulated splicing in animals.

## Abstract (web summary)

Spinal muscular atrophy (SMA) is associated with minor splicing-related defects. Here the authors develop *Drosophila* models with minor spliceosomal-snRNA deletions, and demonstrate SMA-like phenotypes.

## Introduction

RNA splicing removes intronic sequences from the newly transcribed pre-RNA by the spliceosome, a dynamic multi-megadalton RNA-protein complex comprising five small nuclear RNAs (snRNAs) and more than 100 proteins^1–3^. Two types of spliceosomes, the major and the minor, coexist and are both essential in higher eukaryotes^4–7^.

The major spliceosome consists of U1, U2, U4, U5, and U6 snRNAs and is responsible for removing >99.5% of introns (U2-type), while the rest of introns (U12-type) are removed by the minor spliceosome, which consists of U11, U12, U4atac, U5, and U6atac snRNAs^8–11^. Most protein components are shared by the two spliceosomes, except for 65K, 59K, 48K, 35K, 31K, 25K, and 20K, which are unique to the U11/U12 di-snRNP of the minor spliceosome^12,13^. U12-type introns were originally identified to have noncanonical AT-AC splice sites (SSs)^9,10^, and later characterized as having canonical SSs as well^14^. The consensus sequences of the 5′SS, branch site (BS), and the 3′SS, are more conserved than those in U2-type introns^15–17^.

Many human diseases have been linked to the minor spliceosome and U12-type introns^18^. For example, mutations in the 65K protein, U12, and U4atac snRNAs have been identified in growth hormone deficiency, early-onset cerebellar ataxia, and microcephalic osteodysplastic primordial dwarfism type I, respectively^19–21^; and mutations in U12-type introns, the 5′SSs of *STK11* and *TRAPPC2* genes, have been connected with Peutz-Jegher’s syndrome and spondyloepiphyseal dysplasia tarda, respectively^22,23^.

Spinal muscular atrophy (SMA) is proposed to be a minor splicing-related disease^24,25^, which displays severe degeneration of motor neurons in the spinal cord, leading to progressive muscle weakness, mortality and paralysis^26^. The most common types of SMA are caused by mutations in the survival of the motor neuron 1 (*SMN1*) gene on chromosome 5^27,28^. SMN protein is critical for the assembly of small nuclear ribonucleoproteins (snRNPs) that are fundamental components of the two spliceosomes^29^. In humans, *SMN1* gene encodes ~90% of the functional SMN protein, and a paralog, the *SMN2* gene, produces much less protein due to alternative splicing (AS) that skips exon 7^30^; however, many other eukaryotes, including mouse and fly, have only one copy. In SMA models, deficiency of SMN protein results in altered stoichiometry of both the major and minor snRNAs^25,31,32^ and widespread changes of pre-mRNA splicing^33–36^. Although the majority of retained introns are U2-type introns, U12-type introns are proposed to be more sensitive to *SMN* mutations, because a larger proportion of U12-type introns are retained^31,33,34^. However, above mentioned mutations in the human minor-spliceosomal components do not cause SMA, but rather cause other diseases that have completely different phenotypes from SMA.

In *Drosophila* SMA models, *Smn* mutant and RNAi strains have reduced muscle size, motor rhythm, and motor neuron neurotransmission, and result in hindered locomotion^24,37–41^. Restored expression in the motor circuit of *Stasimon*, a U12 intron-containing gene, has been reported to correct defects in neuromuscular junction (NMJ) transmission and muscle growth in *Drosophila Smn* mutants^24^, whereas other studies show that the retention of the *Stasimon* U12 intron is instead a consequence of the developmental arrest^35^.

Since SMN is fundamental to the assembly of both the major and minor spliceosomes, it remains unclear whether defective minor splicing suffices to induce SMA. In addition, genome-wide U12-type introns have not yet been sufficiently investigated. The U12DB website currently lists 695, 555, 306, and 16 of U12-type introns for *H. sapiens*, *M. musculus*, *A. thaliana* and *D. melanogaster*, respectively^42,43^. Recently, ~150 new U12-type introns were added on the Minor Intron DataBase^44^; however, most of them are predicted based on the human consensus sequences^8,45^.

To address the above understanding deficits, we generated minor-spliceosomal-snRNA deletion strains (*U12*^*Δ/Δ*^ and *U6atac*^*Δ/Δ*^) by CRISPR/Cas9 system in *Drosophila* and identified transcriptome-wide sensitive SSs and introns. We then screened sensitive intron-containing neural genes and found three that directly rescue SMA-associated phenotypes, including impaired muscle development, decreased neuromuscular connections, and hindered locomotion. Further analyses revealed a new mode of splicing regulation of minor sensitive introns by competition between the minor and the major spliceosomes. These results demonstrate that the defective minor spliceosome is a major force leading to SMA-associated phenotypes.

## Results

### Precise deletion of minor-spliceosomal snRNAs results in *Drosophila* lethality and defective splicing of U12-type introns

**Fig. 1:**
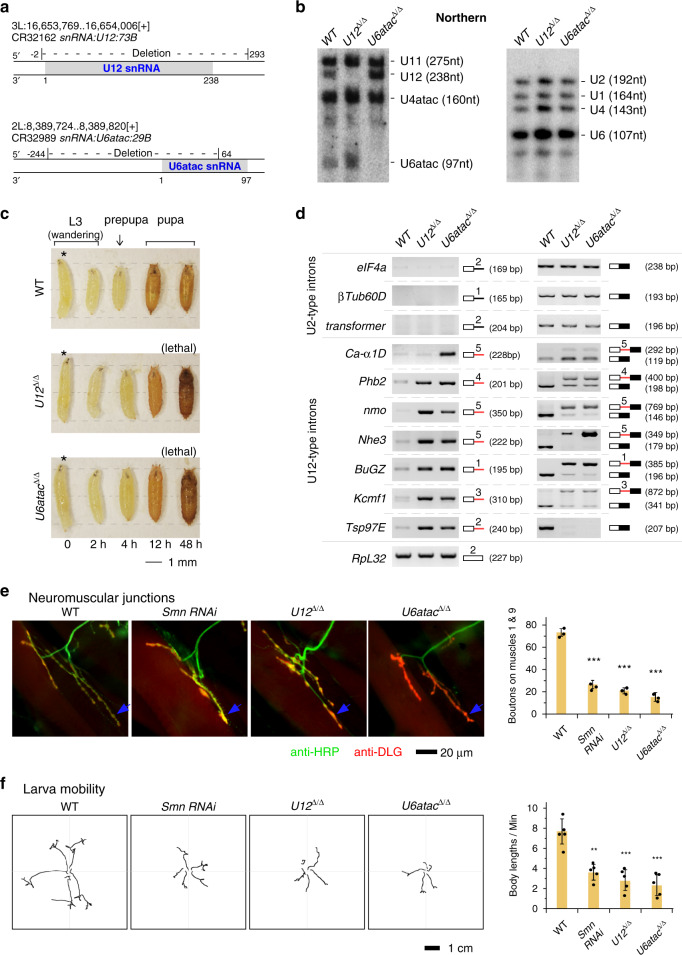
Precise deletion of U12 and U6atac snRNAs by CRISPR-Cas9 system results in defective splicing of U12-type introns and SMA phenotypes in *Drosophila*. **a** Deletion of U12 snRNA and U6atac snRNAs in *Drosophila* by CRISPR/Cas9 system. Gene loci and deleted regions are indicated. **b** Validation of deletion strains by northern blotting. Positions and sizes of the U2- and U12-type snRNAs are indicated. **c** Both the *U12*^*Δ/Δ*^ and *U6atac*^*Δ/Δ*^ strains are lethal at the pupal stage. Pictures of deletion and *WT* strains are shown from L3 wandering to pupal stages. **d** Splicing of U12-type introns are inhibited in deletion strains. Two sets of amplification primers of RT-PCR were used for each intron analysis. Amplification regions are indicated; black lines: U2-type introns; red lines: U12-type introns; boxes: exons. Analyses of other U2- and U12-type introns are shown in Supplementary Fig. 1. **e** Decreased neuromuscular connection in the *U12*^*Δ/Δ*^ and *U6atac*^*Δ/Δ*^ strains. Green: neuron visualized by HRP antibody; red: presynaptic and posterior membranes visualized by DLG antibody. Boutons of NMJ in the colocalized regions are counted under microscopy (*Smn RNAi*: *p* = 0.00016; *U12*^*Δ/Δ*^*: p* = 0.00013; *U6atac*^Δ/Δ^: *p* = 9.3e−5). **f** Hindered larval locomotion of the *U12*^Δ/Δ^ and *U6atac*^*Δ/Δ*^ strains (*Smn RNAi*: *p* = 0.00101; *U12*^Δ/Δ^: *p* = 0.00036; *U6atac*^Δ/Δ^: *p* = 0.00019). Path lengths of larval locomotion are recorded and normalized to body lengths. Data represent the mean ± SD from five representatives of each strain, **p < 0.01, ***p < 0.001. *p* values were calculated using two-sided *t*-test. Source data are provided as a Source Data file.

We first constructed two *Drosophila* strains with precise deletion of minor spliceosome-specific U12 and U6atac snRNAs by the CRISPR/Cas9 system (Fig. 1a and Supplementary Fig. 1a, b). The homozygous strains, *U12*^Δ/Δ^ and *U6atac*^Δ/Δ^, were further confirmed by Northern blotting, showing no detectable U12 or U6atac snRNA (Fig. 1b). The heterozygous strains (*U12*^+/Δ^ and *U6atac*^+/Δ^) appeared to grow normally; however, the homozygous strains are lethal at pupal stages (Fig. 1c), indicating that snRNA components of the minor spliceosome are essential for fly development. In addition to the sixteen U12-type introns listed on U12DB, three more have been reported due to the similarity of their 5′SS with human U12-type introns^42^. Splicing of all these 19 introns was inhibited in the two snRNA deletion strains, whereas splicing of tested U2-type introns remained the same (Fig. 1d and Supplementary Fig. 1c), demonstrating that the two deletion strains have serious defects specific in minor splicing but not in major splicing.

### *U12*^Δ/Δ^ and *U6atac*^Δ/Δ^ strains exhibit SMA-associated phenotypes similar to the *Smn* mutant

To examine if disruption of minor splicing alone can lead to defects in NMJ, we performed immunohistochemistry to visualize neurons by horseradish peroxidase (HRP) antibody, and presynaptic and posterior membranes by disc-large (DLG) antibody on larva muscles of *Smn* RNAi, *U12*^Δ/Δ^ and *U6atac*^Δ/Δ^ strains, respectively. Consistent with previous reports^24,38–40^, our *Smn* RNAi strain also exhibited morphological defects and fewer boutons of NMJ in comparison to the *WT* strain, indicating that it is a useful control for our studies. Interestingly, the NMJ bouton numbers of the *U12*^*Δ/Δ*^ and *U6atac*^*Δ/Δ*^ strains were around 70% less than the *WT* strain, indicating defective neuromuscular connections in all three strains (Fig. 1e). Next, we evaluated the motion capability of fly strains. The larval locomotion distance of *Smn* RNAi, *U12*^*Δ/Δ*^ and *U6atac*^*Δ/Δ*^ strains was significantly shorter than the *WT* strain (Fig. 1f), thus indicating an impaired behavior due to the defective NMJ.

Altogether, these data demonstrate that the deletion of minor-spliceosomal snRNAs results in SMA-associated phenotypes similar to the *Smn* mutant, providing direct evidence that these phenotypes result from disruption of minor spliceosomes.

### Identification of minor spliceosome-sensitive introns and SSs

**Fig. 2:**
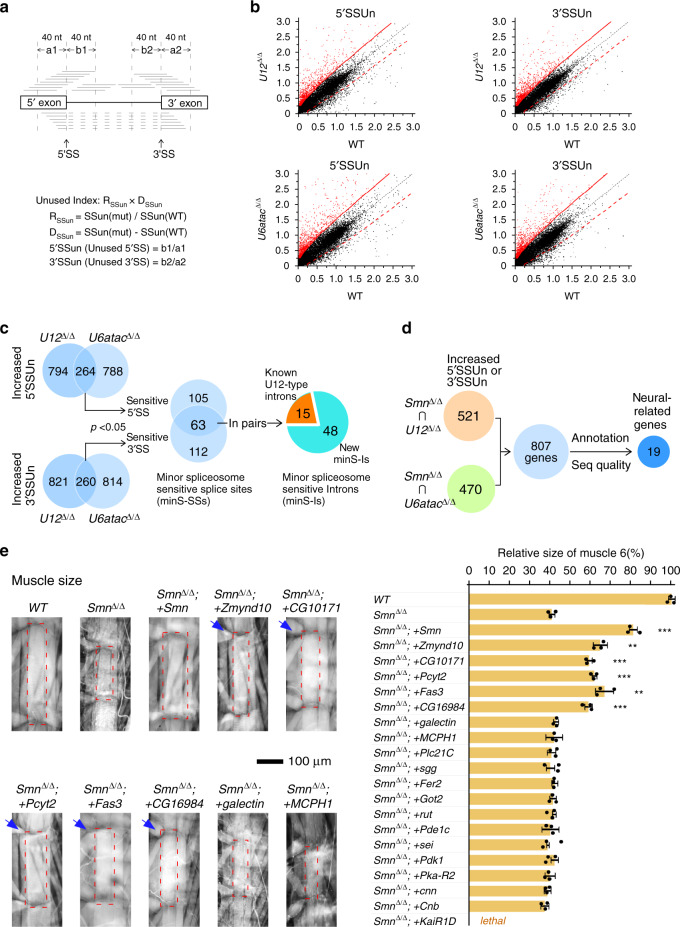
Identification of minor spliceosome-sensitive splicing events and candidate genes for the rescue of SMA-associated phenotypes. **a** Diagram of bioinformatic analyses for identifying minS-Is. For details see methods. **b** Distribution of SSun scores of transcriptome-wide 5′SSs and 3′SSs in the mutant strain (*U12*^Δ/Δ^ or *U6atac*^Δ/Δ^) versus the *WT* strain. Grouped by a red unbroken limitation line, red dots represent the top 2% of SSun scores in the mutant strains; and black dots represent the remaining SSun scores, where a symmetric red dashed control line is indicated. **c** Splice sites and introns that are sensitive to the two minor-snRNA deletions. Decreased usage of the 5′ and 3′SSs are identified and paired. minS-Is are grouped into known U12-type introns and new minS-Is. **d** Selection of neural genes with defective 5′SS or 3′SS in both *Smn* deletion and minor-snRNA deletion strains. **e** Development of muscle 6, indicated within the dashed red rectangles, is rescued by five neural genes. Positive genes are indicated by blue arrow; additional transgenic strains with neural expressed CDSs of candidate genes are presented in Supplementary Fig. 4b (*Smn*^Δ/Δ*;*^ + *Smn*: *p* = 2e−5; *Smn*^Δ/Δ*;*^ + *Zmynd10*: *p* = 0.00281; *Smn*^Δ/Δ;^ + *CG10171*: *p* = 8.5e−5; *Smn*^Δ/Δ;^ + *Pcyt2*: *p* = 5.5e−5; *Smn*^Δ/Δ;^ + *Fas3*: *p* = 0.00583; *Smn*^Δ/Δ;^ + *CG16984*: *p* = 0.00025). Data represent the mean ± SD from five representatives of each strain. **p < 0.01 and ***p < 0.001. *P* values were calculated using two-sided *t*-test. Source data are provided as a Source Data file.

To address how splicing and introns were affected transcriptome-wide in the deletion strains, we performed RNA-seq of L3 larvae of the *WT*, *U12*^Δ/Δ^, and *U6atac*^Δ/Δ^ strains. Approximately 60 M paired-end reads from each sample were mapped to the *Drosophila* genome (Supplementary Data 1). By using *rMATS*, hundreds of AS events were found to be significantly changed (Supplementary Fig. 1d), however, this method only identified a small portion of U12-type introns, as pointed out by a previous report^35^. To improve the detection sensitivity and accuracy, we developed a new bioinformatic approach to measure the transcriptome-wide “Unused Index” of all SSs based on the ratio of reads between the 40-nt intronic and exonic regions flanking each SS (Fig. 2a and details in “Methods”). According to preliminary RT-PCR results, we selected the top 2% of SSs on the Unused Index for further analyses (Fig. 2b and Supplementary Data 2). The use of 168 5′SSs and 175 3′SSs was significantly decreased in both *U12*^Δ/Δ^ and *U6atac*^Δ/Δ^ strains, and 63 5′SSs and 3′SSs were in pairs from the same introns, which we designated as minor-spliceosome-sensitive splice sites (minS-SS) and introns (minS-I), respectively (Fig. 2c). Among the 63 minS-Is, 15 are known as U12-type introns and 48 are new (Supplementary Data 3). To validate these findings, ten new minS-Is were randomly picked and tested by RT-PCR; their splicing was clearly inhibited in the two deletion strains (Supplementary Fig. 1e), consistent with our bioinformatic analyses (Supplementary Fig. 1f). Thus, in addition to classic U12-type introns, we identified new minS-Is and many minS-SSs in *Drosophila* through transcriptome analyses of minor snRNA deletion strains.

### Selection of candidate genes for rescue of SMA-associated phenotypes

Considering the level of knockdown based on RNAi was not stable and insufficient, we generated another CRISPR/Cas9-mediated deletion strain, *Smn*^Δ/Δ^ (Supplementary Fig. 2a), which was used in a confirmatory approach for screening candidate genes that would directly contribute to SMA-associated phenotypes. The *Smn*^Δ/Δ^ strain exhibited stronger phenotypes than the *Smn* RNAi strain. Unused index analysis of this *Smn*^Δ/Δ^ strain indicated that it had more affected SSs than in *U12*^Δ/Δ^ and *U6atac*^Δ/Δ^ strains (Supplementary Fig. 2b). To address whether the *Smn*^Δ/Δ^ strain was proper for the SMA-associated genetic screen, we performed a time course detection of NMJ for the *Smn*^Δ/Δ^ strain (Supplementary Fig. 2c, d). In comparison to the *WT* strain, amounts of NMJ in the *Smn*^Δ/Δ^ larva were at similar levels in their first 3 days to the time point 96 h post egg-laying. After that, we observed strongly decreased NMJ in the *Smn*^Δ/Δ^ strain, demonstrating that degeneration of motor neurons occurred in the *Smn*^*Δ/Δ*^ strain after L3_24 hr. Through analyzing a large set of *Drosophila* RNA-seq data, including the modEncode Drosophila data sets^46^ and *Smn* mutant^35^, the developmental stage of the *Smn*^*Δ/Δ*^ strain was determined as after the L3_12 hr, while the *Smn* RNAi, *U12*^*Δ/Δ*^ and *U6atac*^*Δ/Δ*^ strains were grouped with the *WT* larva samples at the stage of L3_PS1-2 (Supplementary Fig. 2e). Further characterizations indicated that our CRISPR-Cas9 generated strains, *U12*^Δ/Δ^, *U6atac*^Δ/Δ^ and *Smn*^Δ/Δ^, did not have detectable off-target effects (Supplementary Fig. 3a–d).

Comparing Unused indexes of SSs from the three deletion strains, we identified 521 genes that had at least one 5′SS or 3′SS with significantly decreased usage shared between the *Smn*^Δ/Δ^ and *U12*^Δ/Δ^ strains, and 470 genes by the *Smn*^Δ/Δ^ and *U6atac*^Δ/Δ^ strains, which led to 807 genes in total (Fig. 2d). After manual inspection of RNA-seq signals and gene annotations, nineteen neural-related genes were selected as candidates for rescue screens (Supplementary Data 4).

### Rescue of SMA-associated phenotypes by expression of minS intron-containing neural genes

We screened candidate genes through the construction of transgenic flies in the *Smn*^Δ/Δ^ background, where each CDS was driven by neural-specific *elav*^*C155*^-*Gal4* through crossing *Smn*^*+/Δ*^ heterozygotes due to *Smn*^Δ/Δ^ lethality. Except for one strain with the *KaiR1D*-CDS that died at the embryonic stage, phenotypes of other strains were tested.

First, body size of *Smn*^Δ/Δ^ larvae was much smaller than that of the *WT*, and was rescued by adding back CDSs of *Smn* and five candidate genes, including *Zmynd10*, *CG10171*, *Pcyt2*, *Fas3* and *CG16984*, whereas 14 others had little effect (Supplementary Fig. 4a). Further, neural expression of the above five genes’ CDSs significantly restored the reduced muscle size (muscle 6 here) caused by *Smn*^Δ/Δ^, showing restoration of muscle development, whereas 14 other genes did not (Fig. 2E and Supplementary Fig. 4b).

**Fig. 3:**
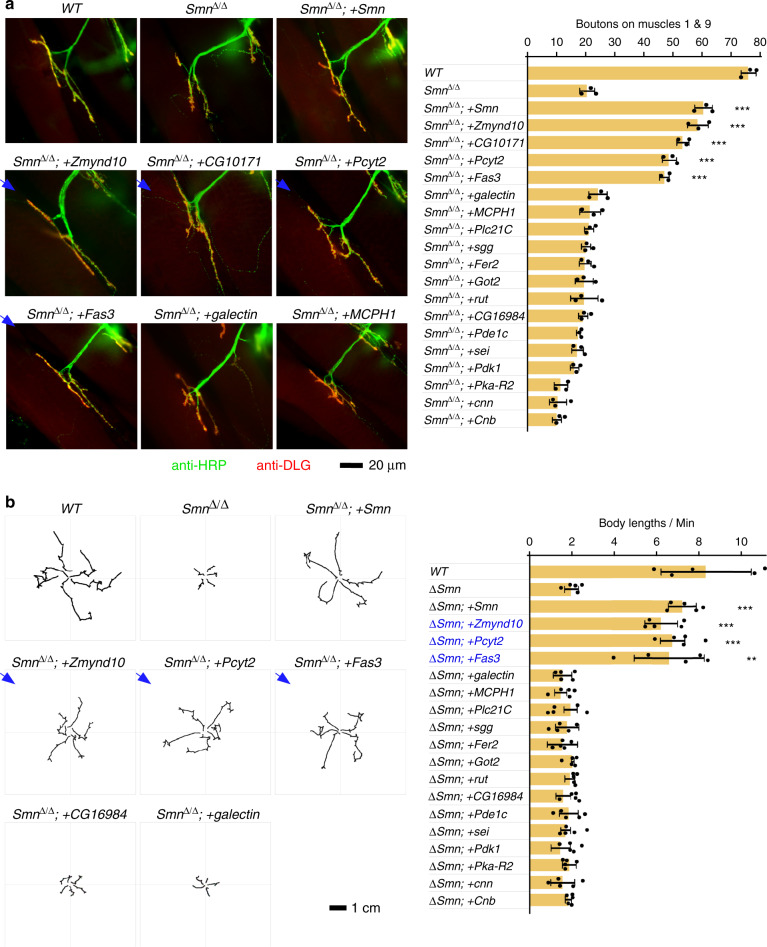
Screen of neural genes that could rescue NMJ defects and larvae mobility. **a** Left, immunostaining of neuromuscular connections in transgenic strains driven by *elav-GAL4* in the *Smn*^Δ/Δ^ background. NMJ boutons are counted and the additional transgenic strains are shown in Supplementary Fig. 5a. Right, quantitation of boutons in each strain (*Smn*^Δ/Δ*;*^ + *Smn*: *p* = 8.3e-5; *Smn*^Δ/Δ;^ + *Zmynd10*: *p* = 0.00021; *Smn*^Δ/Δ*;*^ + *CG10171*: *p* = 8.3e−5; *Smn*^Δ/Δ;^ + *Pcyt2*: *p* = 0.00017; *Smn*^Δ/Δ;^ + *Fas3*: *p* = 0.00025). **b** Left, larval locomotion of transgenic strains (*Smn*^Δ/Δ;^ + *Smn*: *p* = 8.1e−6; *Smn*^Δ/Δ;^ + *Zmynd10*: *p* = 0.0001; *Smn*^Δ/Δ;^ + *Pcyt2*: *p* = 9.3e−5; *Smn*^Δ/Δ;^ + *Fas3*: *p* = 0.00442). Additional transgenic strains are shown in Supplementary Fig. 5d. Right, quantitation of path length of larval locomotion. Data represent the mean ± SD from five representatives of each strain. **p < 0.01, ***p < 0.001. *P* values were calculated using two-sided *t*-test. Source data are provided as a Source Data file.

Second, the *Smn*^Δ/Δ^ strain exhibited dramatically fewer NMJ boutons as expected, and the defects were rescued by adding back the *Smn*-CDS (Fig. 3a). Among the candidates, four of them, *Zmynd10*, *CG10171*, *Pcyt2*, and *Fas3*, significantly restored the NMJ, but others could not (Fig. 3a and Supplementary Fig. 5a). Although the *CG16984-*CDS rescued the body size and muscle development, it could not rescue the NMJ defects caused by *Smn*^Δ/Δ^, suggesting the function of *CG16984* may be limited to muscle cell proliferation^47^. To examine if the rescue effect of NMJ by the four genes was specific to SMN ablation, we expressed these genes in *WT Drosophila* and observed that neural-specific expression of *CG10171* led to the increase of the NMJ boutons, whereas *Zmynd10*, *Pcyt2* or *Fas3* had no effect (Supplementary Fig. 5b, c). These results demonstrated that the restoration of neuromuscular connections in *Smn*^*Δ/Δ*^ by the expression of *Zmynd10*, *Pcyt2*, and *Fas3* are reliable, and *CG10171* may work on certain SMN independent pathway to regulate NMJ boutons.

Lastly, the impaired larval locomotion caused by *Smn*^*Δ/Δ*^ was significantly rescued by neural expression of *Zmynd10*, *Pcyt2*, and *Fas3*, showing similar levels of restoration as the *Smn*-CDS did, whereas other CDSs did not (Fig. 3b and Supplementary Fig. 5d). In sum, after inspection of four different phenotypes, we conclude that three neural genes, *Zmynd10*, *Pcyt2*, and *Fas3*, rescue SMA-associated phenotypes caused by SMN deficiency.

### *Pcyt2*, *Zmynd10*, and *Fas3* are targets of the minor spliceosome

Neural-related functions of the above three genes have been previously investigated. Specifically, *Pcyt2* (phosphocholine cytidylyltransferase 2), together with *Pcyt1* in *Drosophila*, are responsible for the regulation of phosphatidylcholine in neural membranes^48,49^; *Zmynd10* (zinc finger MYND-type containing 10) is essential for proper axonemal assembly of Dynein arms, and its loss-of-function mutant exhibits uncoordinated locomotion due to chordotonal neuron malfunction^50^. *Fas3* (fasciclin III) is a cell adhesion molecule, expressed in motor neurons and their synaptic target muscle cells during neuromuscular development^51^.

**Fig. 4:**
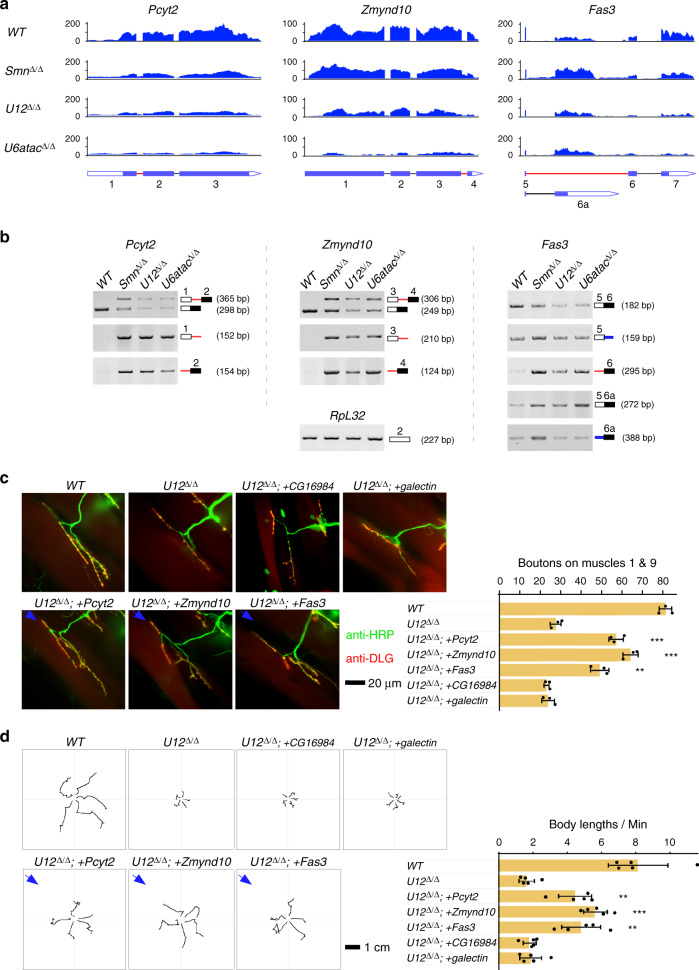
*Pcyt2*, *Zmynd10*, and *Fas3* are targets of the minor spliceosome. **a** RNA-seq signals of the three genes in the four *Drosophila* strains. minS-Is are indicated. **b** Splicing of minS-Is of the three genes are significantly inhibited in deletion strains. Multiple sets of primers for each intron are used for RT-PCR. Red lines: minS-Is; black lines: U2-type introns; blue lines: mixture of the minor and major spliceosomes recognized introns. **c** Decreased NMJ boutons are rescued by neural expression of the three genes in *U12*^*Δ/Δ*^ background (*U12*^Δ/Δ;^ + *Pcyt2*: *p* = 0.00059; *U12*^Δ/Δ;^ + *Zmynd10*: *p* = 0.00028; *U12*^Δ/Δ;^ + *Fas3*: *p* = 0.00428). **d** Impaired larval locomotion caused by *U12*^Δ/Δ^ are improved by neural expression of the three genes (*U12*^Δ/Δ;^ + *Pcyt2*: *p* = 0.0023; *U12*^Δ/Δ;^ + *Zmynd10*: *p* = 2.9e−5; *U12*^Δ/Δ;^ + *Fas3*: *p* = 0.00324). Data represent the mean ± SD from five representatives of each strain. **p < 0.01, ***p < 0.001. *p* values were calculated using two-sided *t*-test. Source data are provided as a Source Data file.

Based on our transcriptome analyses, the use of the 5′ and 3′SSs of *Pcyt2-*intron 1 and *Zmynd10-*intron 3, and the 3′SS of *Fas3-*intron 5 was significantly decreased in the deletion strains (Fig. 4a and Supplementary Data 4), those splicing defects could result in altered protein levels and isoforms (Supplementary Fig. 6). Further RT-PCRs revealed that *Pcyt2-*intron 1 and *Zmynd10-*intron 3 were severely retained in the *Smn*^Δ/Δ^, *U12*^Δ/Δ^, and *U6atac*^Δ/Δ^ strains (Fig. 4b), demonstrating that their splicing inhibition is due to the defective minor spliceosome. There is an alternative 3′SS inside of the *Fas3-*intron 5, and use of this 3′SS generates another mRNA isoform that contains exon 6a instead of exon 6 (Fig. 4a right). Using multiple sets of amplification primers, RT-PCRs revealed that splicing of *Fas3-*intron 5 was clearly inhibited in the three deletion strains, showing increased retention of intron 5 and decreased level of exon 6-containing mRNA, while the alternative exon 6a-containing mRNA product was increased (Fig. 4b right). This result suggests that the *Fas3-*intron 5 comprised of the common 5′SS and the minS-3′SS is recognized by the minor spliceosome, whereas the intron comprised of the common 5′SS and the alternative 3′SS is recognized by the major spliceosome. A defective minor spliceosome results in increased recognition of the common 5′SS by the major spliceosome. In addition, from data of RNA-seq and RT-PCRs, we observed that expression levels of these three genes in the *U12*^Δ/Δ^ and *U6atac*^Δ/Δ^ strains are lower than their levels in the *Smn*^Δ/Δ^ strain, this might due to various levels of RNA surveillance or other unknown reasons.

We then asked whether these genes could rescue SMA-associated phenotypes caused by *U12*^*Δ/Δ*^. Neural-specific expression of *Pcyt2-*, *Zmynd10-*, and *Fas3-*CDSs clearly rescued the defective neuromuscular connections and impaired locomotion in *U12*^Δ/Δ^ background, exhibiting increased NMJ boutons (Fig. 4c) and longer paths of larvae movement (Fig. 4d), whereas two controls, *CG16984* and *galectin*, did not. Taken together, these data demonstrate that *Pcyt2*, *Zmynd10*, and *Fas3* are effectors of SMA-associated phenotypes caused by both *Smn* mutant and defective minor spliceosomes.

### minS-Is are in general associated with minor-spliceosomal components

**Fig. 5:**
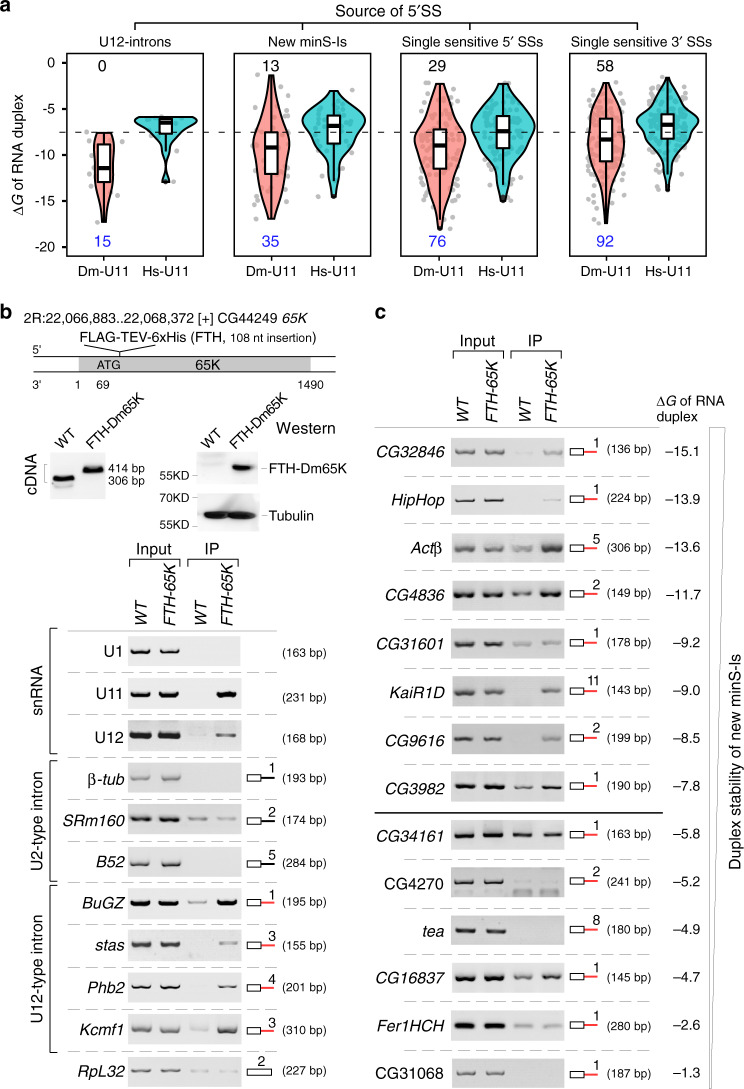
Association of new minS-Is with minor-spliceosomal components. **a** Predicted stability of RNA duplexes formed by the 5′SS of minS-Is and U11 snRNA. Potential base-pairs and free energy are calculated by *RNAduplex*; both *Drosophila* and human U11 snRNAs are used for duplex analyses. *n* = 15 (U12 intron); *n* = 48 (new minS-Is); *n* = 105 (single sensitive 5′SSs); *n* = 150 (single sensitive 3′SSs). **b** Dm65K is a specific component of the minor spliceosome. The *FTH-Dm65K* strain constructed using the CRISPR/Cas9 system is validated by PCR, sequencing, and western blot (upper). FTH-Dm65K enriches minor snRNAs and U12-type introns, but not major snRNA or U2-type introns (lower). Co-purifications were performed using lysate from the WT and *FTH-Dm65K Drosophila* strains. **c** Association of new minS-Is with Dm65K. FTH-Dm65K enriches pre-mRNA of most new minS-Is that contain 5′SS predicted to form a stable duplex with U11 snRNA. minS-Is (red line) and free energy (Δ*G*) are indicated. In (**a**) boxplots, the middle line show data median, the lower and upper hinges correspond to the 25th and 75th percentiles, the upper whisker extends from the hinge to the largest value no further than 1.5  × IQR from the hinge (where IQR is the interquartile range) and the lower whisker extends from the hinge to the smallest value at most 1.5 × IQR of the hinge. Source data are provided as a Source Data file.

To understand why splicing of the minS-Is and minS-SSs are inhibited in the deletion strains, we first analyzed potential base-pairs between sequences of the 5′SSs (−5 to +20) and the 5′-end of U11 snRNA (+1 to +20), and measured duplex stabilities using *RNAduplex*^52^. All 5′SSs of known *Drosophila* U12-type introns could form stable duplexes with low free energies with *Drosophila* U11 (Dm-U11) (Fig. 5a and Supplementary Data 3), consistent with their prediction based on the consensus sequence. Using the highest free energy of RNA duplex formed by U12-type *CG11839-*intron 1 (Δ*G* = −7.6) as a cut-off, we found that more than 70% of 5′SSs from the minS-Is and minS-SSs could form stable duplexes with the Dm-U11 snRNA, but these same 5′SSs would pair significantly less stably with human U11 snRNA (Hs-U11) (Fig. 5a and Supplementary Data 3). The stabilities of many duplexes are even higher than those containing known U12-type introns (Supplementary Fig. 7a), implying that most of the new minS-Is could be recognized by the minor spliceosome through 5′SS-U11 interaction in *Drosophila*.

This computational prediction prompted us to examine associations of the new minS-Is with the minor spliceosome in vivo. In human U11/U12 di-snRNP, seven minor-spliceosomal-specific proteins were identified^12,13^. However, only two have described homologs in *Drosophila* (Dm65K and Dm20K)^42^. We first transfected a Flag-Dm65K plasmid into *Drosophila* S2 cells and found that Flag-Dm65K specifically co-purified U11 and U12 snRNAs, but not U1 or U2 snRNA (Supplementary Fig. 7b). In the other direction, Flag-Dm65K was efficiently pulled down by anti-SmD2 antibody and by an anti-sense oligo to U11 snRNA (Supplementary Fig. 7c), validating that Dm65K is a component of the *Drosophila* U11/U12 di-snRNP. Second, we constructed a CRISPR/Cas9-mediated “knock-in” fly strain, in which an FTH-tag was inserted into the endogenous N-terminus of Dm65K (Fig. 5b upper). As in S2 cells, FTH-Dm65K enriched U11 and U12 snRNAs, as well as U12-type but not U2-type introns (Fig. 5b lower). Furthermore, 7 out of 8 of tested minS-Is predicted to form stable duplexes with U11 were enriched by FTH-Dm65K, whereas 5 out of 6 of those predicted to not form stable duplexes were not enriched (Fig. 5c), suggesting that most of the newly identified minS-Is are substrates of the minor spliceosome, whereas others might be affected indirectly.

### Alternative selection by the major and minor spliceosomes

**Fig. 6:**
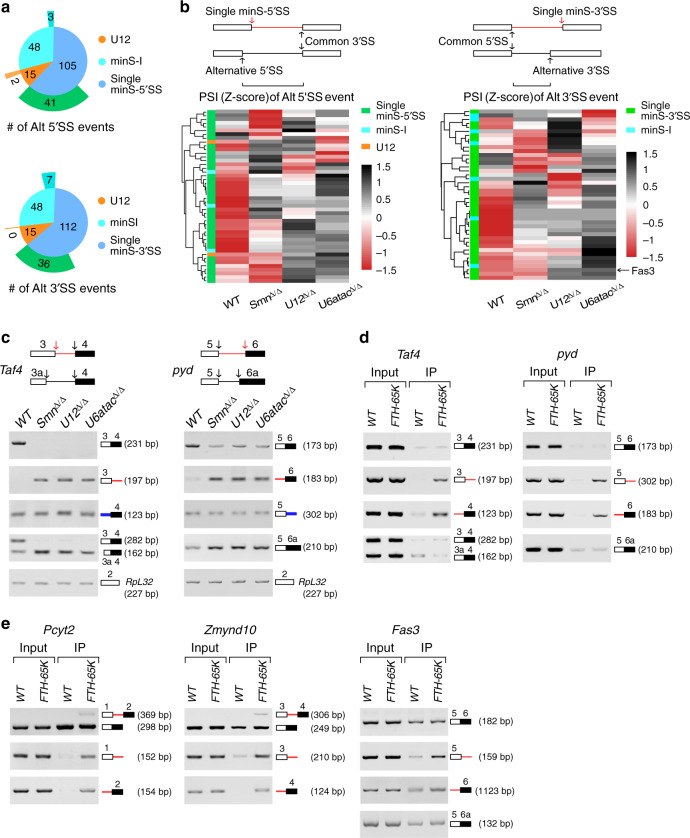
Alternative selection by the major and minor spliceosomes. **a** Alternative splicing events that are involved with minor spliceosome sensitive SSs. Upper, alternative 5′SS events identified from three categories of minS-5′SSs; lower, alternative 3′SS events identified from three categories of minS-3′SSs. **b** Upper, schematics of splice sites in the minS-SS-involved AS events. Lower, splicing of introns using the alternative SSs and common SSs were increased in the minor-snRNA deletion strains. **c** Common SSs can be recognized by both the major and minor spliceosomes. Five sets of primers were used to amplify various pre-mRNAs and mRNAs. **d** Pre-mRNAs but not mRNAs of the minor spliceosome-sensitive genes are enriched by minor-specific Dm65K. **e** Pre-mRNAs of the genetically identified three genes that rescue SMA-associated phenotypes are enriched by Dm65K. *FTH-Dm65K* and the *WT* strains were used. Red arrows and lines: minS-SSs and minS-Is; black arrows and lines: the major spliceosome recognized SSs and introns; blue lines: mixture of the minor and major spliceosomes recognized introns. Source data are provided as a Source Data file.

Including the 3′SS of *Fas3*-intron 5, in total 217 single minS-SSs, either 5′SS or 3′SS of an intron, were identified in the *Drosophila* genome (Fig. 2c). To understand why only a single SS instead of the SS pair is sensitive to the defective minor spliceosomes, we searched transcriptome-wide AS events and found that a large subset of single minS-SSs, 39.0% of the 5′SSs and 32.1% of the 3′SSs, was involved in AS, while the paired minS-SSs from the U12-type introns and newly identified minS-Is were much less likely to be involved in AS (Fig. 6a). For those AS events, there are two possibilities for SS selections: one uses the single minS-SS with the common SS, the other uses the alternative SS(s) with the common SS (Fig. 6b upper). Further analyses of AS changes revealed that splicing of most introns that use the alternative SS with the common SS were increased in the *U12*^Δ/Δ^ and *U6atac*^Δ/Δ^ strains compared to those in the *WT* and *Smn*^Δ/Δ^ strains; in contrast, splicing of introns that use the single minS-SS with the common SS were inhibited in the minor-snRNA deletion strains (Fig. 6b). This pattern was validated by multiple sets of RT-PCRs of tested genes (Fig. 6c and Supplementary Fig. 7d). For example, the 5′SS of *Taf4*-intron 3 was identified as a minS-SS, splicing of intron using this minS-SS with the common 3′SS was totally inhibited in the minor-snRNA deletion strains, while splicing of intron using its alternative 5′SS with the common 3′SS was increased, showing a competition between the two mRNA isoforms (Fig. 6c left). Co-immunoprecipitation (Co-IP) using the *FTH-Dm65K* strain showed that pre-mRNAs, but not mRNAs, of these genes, could be enriched by Dm65K Co-IP (Fig. 6d and Supplementary Fig. 7e), demonstrating that those introns, comprising the single minS-SS and common SS, can be recognized by the minor spliceosome. In contrast, those introns, comprising the alternative SS and common SS, are recognized by the major spliceosome, suggested by their increased splicing in the two minor spliceosome disrupted strains. Thus, these results reveal that many common SSs related to the identified single minS-SSs could be recognized by both the major and minor spliceosomes, and alternative selection occurs when the function of the minor spliceosome is disrupted.

**Fig. 7:**
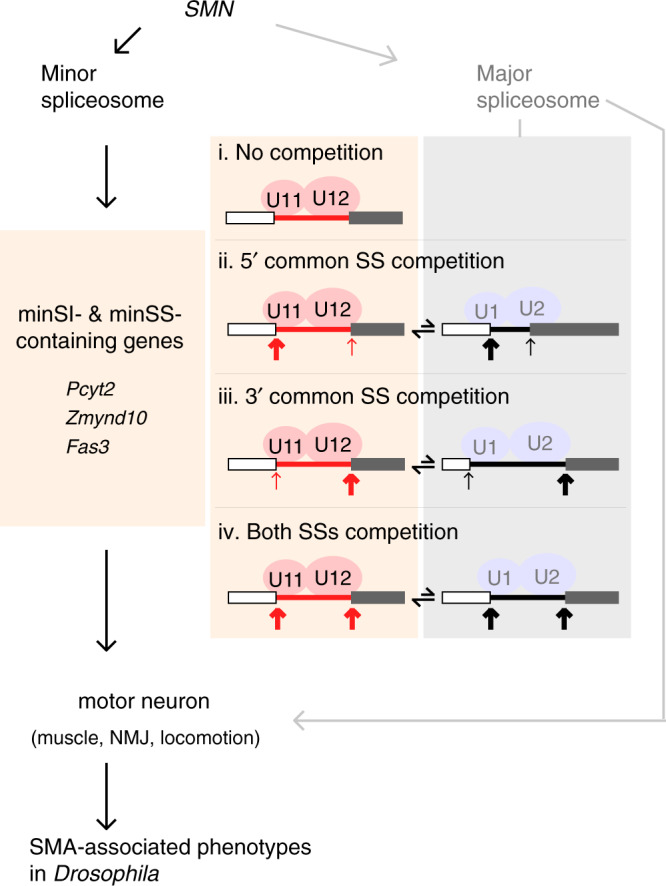
Schematic of defective minor spliceosome induced SMA-associated phenotypes and competition model of minor intron recognition. *SMN* is the upstream gene of SMA; a defective minor spliceosome results in inhibited splicing of minS-I- and minS-SS-containing neural genes, and directly induces SMA-associated phenotypes. Splicing recognition of U12-type or minor sensitive introns (minS-I) are proposed as: (i) only by the minor spliceosome; (ii & iii) 5′SS or 3′SS is competitively recognized by the minor and major spliceosomes; (iv) both SSs are competitively recognized by the two spliceosomes.

In addition, pre-mRNAs of the genes identified above, *Pcyt2*, *Zmynd10*, and *Fas3*, were also enriched by FTH-Dm65K Co-IP (Fig. 6e). We conclude that these three minS intron-containing neural genes are downstream targets of SMN and the minor spliceosome, and are major effectors of the SMA-associated phenotypes (Fig. 7 left).

## Discussion

*SMN* is the upstream gene associated with SMA, mutations/deletions of *SMN* trigger chaos of downstream genes’ expression and splicing, leading to degenerated motor neurons. In this study, we provide several lines of evidence to support that disruption of the minor spliceosome directly induces SMA in *Drosophila*: (1) deletion of minor spliceosome-specific snRNAs results in defective function of motor neurons and reveals many minS-Is and minS-SSs by a newly developed bioinformatic approach; (2) splicing of numerous neuron-related genes are inhibited in the *U12*^Δ/Δ^ and *U6atac*^Δ/Δ^ flies; (3) among them, three minS intron-containing genes can rescue SMA-associated phenotypes in both *Smn*-deleted and minor snRNA-deleted flies (Fig. 7). In addition, AS analyses in minor-snRNA deletion strains reveal competition recognition by the minor and major spliceosomes.

### Defective minor spliceosomes cause developmental defects and suffice to induce SMA-associated phenotypes

Both the *U12*^Δ/Δ^ and *U6atac*^Δ/Δ^ strains that were generated by CRISPR/Cas9 tools are lethal at their pupa stages; this is later than in a previous report of P element-mediated disruptions of the U12 and U6atac genes, which caused lethality during embryonic and the third instar larval stages, respectively^7^. In the beginning of this study, we observed that two of our deletion strains were lethal at the pre-pupal stage, but showed a stable lethal phenotype at the pupation stage when the deletion strains were constructed after crossing with the isogenic *WT* strain for 10 generations.

At the larvae stage, these two deletion strains exhibited strong motor neuron-related defects, fewer NMJ and impaired larva locomotion, similar to the *Smn* mutation/deletion flies (Fig. 1). SMN is critical for the assembly of both the major and minor snRNPs^29^, therefore, mutations/deletions of the *SMN* gene affects both major and minor splicing^31,33^. The similarity of SMA-associated phenotypes between the *Smn* mutants and the minor-snRNA null mutants strongly suggest that these phenotypes caused by the *Smn* mutations could be due to defective splicing of minor-spliceosomal introns and their downstream genes.

### Competition between the minor and major spliceosomes

Most currently known U12-type introns are predicted based on consensus sequences from dozen human U12-type introns, therefore, introns that are recognized by the minor spliceosome would be underestimated without transcriptome-wide investigation, especially in other species. Splicing changes are normally analyzed by software *rMATs* or *MISO*^53^, which mostly rely on the calculation of changes of the exon-exon junction reads that define introns by paired 5′SS and 3′SS. However, the pairs of SSs are variable in AS events, resulting in difficulties to distinguish whether an affected intron (or exon) is due to the defective minor spliceosome or not. In this study, we performed RNA-seq and analyzed transcriptome data using a new approach, in which an “Unused Index” for each SS in the *Drosophila* genome was introduced. This new method avoids interference from routine analyses of AS events and identified 48 new minS-Is and 217 single minS-SSs as well as known U12-type introns. Although the new minS-Is don’t share the conserved consensus sequences with U12-type introns (Supplementary Fig. 7f), most of their 5′SSs are predicted to form a stable duplex with the 5′-end of U11 snRNA and were enriched in co-IP by the minor-spliceosomal component Dm65K (Fig. 5).

Suggested by total inhibition of splicing in the minor-snRNA deletion strains, many U12-type introns and newly identified minS-Is are recognized specifically by the minor spliceosome only (i in Fig. 7). Interestingly, AS changes of the single minS-SSs-involved events reveal that their common SSs can be recognized by both the major and minor spliceosomes, suggesting a competition mechanism for splicing regulation of minor sensitive introns, through either the 5′ common SS competition or the 3′ common SS competition (ii and iii in Fig. 7). In the *WT* strain, the common SSs favor recognition by the minor spliceosome, and result in productive splicing of introns with minS-SSs. However, in the minor spliceosome disrupted strains, the common SSs are recognized by the major spliceosome, and result in productive splicing of introns with alternative SSs (Fig. 6c and Supplementary Fig. 7d). In addition, we observed that several classical U12-type introns, such as those in *Ca-α1D*, *Phb2*, *BuGZ*, and *Kcmf1-a* genes, were partly spliced in the minor spliceosome disrupted strains (Fig. 1d), indicating that both of their two SSs might be recognized by the major spliceosome when the minor spliceosome is disrupted (iv in Fig. 7). We conclude that splicing of many minor sensitive-introns are regulated in a competition mode between the minor and major spliceosomes.

We also analyzed a published transcriptome dataset of human neuron-related SH-SY5Y cells, in which expression of *SMN1* was knocked down through RNAi^33^, and found that stabilities of the 5′SS-U11 RNA duplexes formed by 5′SSs from the 6,176 retained introns are statistically more stable than those from the non-retained introns (Supplementary Fig. 8a), implying that the human system may also have minS-Is and minS-SSs analogous to those we found in *Drosophila*.

### Downstream effectors of SMA

In *Drosophila*, we identified *Pcyt2*, *Zmynd10*, and *Fas3* as downstream targets of *Smn* and demonstrated that their add-back suppressed the SMA-associated phenotypes. Their human homologs are *PCYT1A*, *ZMYND10*, and *Cadm4*, respectively. Mutations of these three genes have been reported with motor neuron-related diseases, such as spondylometaphyseal dysplasia with cone-rod dystrophy caused by mutations in human *PCYT1A* gene^54,55^, primary ciliary dyskinesia-22 caused by mutations in human *ZMYND10* gene^56^. Mice with deletion of *Cadm4* develop focal hypermyelination resulting in abnormal axon-glial contact and redistribution of ion channels along the axon^57^. Taken together, these three genes are critical in the development of motor neurons in Drosophila, mouse and human, as well as harboring a minor-spliceosome-sensitive intron.

In addition, human *Pcyt2* is another homolog of *Drosophila Pcyt2* and contains a U12DB-listed U12-type intron^58^. Data analyses from the *SMN1* knocked-down SH-SY5Y cells showed that each of the above four human genes has a retained intron (Supplementary Fig. 8b), suggesting that *Pcyt2*, *Zmynd10*, and *Fas3*, are conserved across species as affected downstream targets of *Smn* mutants and effectors of SMA. We notice that the SMA models and phenotypes in *Drosophila* are different from in human disease. The majority of infants with infantile-onset SMA are viable; however, *Smn*, *U12,* and *U6atac* mutant flies are lethal at the larval or pupal stage. This is most likely due to the large scale reconstruction of organisms during insect metamorphosis, which are non-neural related.

Recently, two therapeutic methods have been approved for SMA patients by the FDA. The first is Spinraza, a medicine designed based on antisense oligos that enhance splicing inclusion of exon 7 in the *SMN2* gene to generate sufficient SMN protein to compensate for mutation of the *SMN1* gene^59,60^. The second is Zolgensma, a replacement therapy for the mutated *SMN1* gene^61^. Our new method and identified minS intron-containing genes presented in this study would be helpful for future diagnosis and gene therapy of SMA.

## Methods

### Fly strains and culture

The wildtype used in this study is a *w*^*1118*^ isogenic strain (BDSC 5905). Strains *Df(2L)ED629*, *Df(3L)BSC561* and *smn*^*f05960*^ were all obtained from BDSC^39,62,63^. Deletion strains were constructed using CRISPR/Cas9 system^64^. In brief, target sequences of two guide RNAs (sgRNA) were selected, and each pair of gRNA plasmids were co-injected into embryos of the transgenic line nanos-Cas9 by UniHuaii Technology Company. Primers located outside the deletion regions were used for genomic PCRs to screen for the desired alleles, which were further validated by sequencing. The flies obtained were then crossed for at least five generations with the wildtype strain to eliminate potential off-target events and they were finally balanced over *CyO* or *TM3* with GFP in order to distinguish the homozygotes during larva stages. The *FTH-Dm65K* strain was constructed using “knock-in” strategy similar to the deletion strains with co-injection of an additional donor plasmid (pMD18-T) with FTH-insertion and the adjacent 3 kb sequences as homologous arms.

*da-Gal4* and *elav*^*C155*^-*Gal4* strains were used as ubiquitous and neuron-specific drivers, respectively^65,66^. *UAS-Smn*^*shRNA*^ (TH02847.N) was from Tsinghua Fly Center; *UAS-sgg* (no. 5435) and *UAS-rut* (no. 9405) were from the Bloomington Drosophila Stock Center. CDSs of candidate genes were cloned into pBID-UASC^67^ and integrated at the attP40 site on chromosome 2L using phiC31 System^68^. All constructed strains were confirmed by PCR and sequencing.

For rescue, *UAS-gene(CDS); Smn*^*Δ*^*/Tm3, Ser, GFP* males were crossed with female virgins of *elav*^*C155*^-*Gal4*; *Smn*^*Δ*^*/Tm3, Ser, GFP*. They were cultured in egg-laying cages with change of the sucrose agar-coated yeast paste every 12 h. The embryos were cultured on the plates supplied with yeast paste at 25 °C^38^. After 3 and 5 days post egg-laying, the heterozygotes with “green balancers” were picked out twice and discarded in order to reduce the competition between larvae. After 6 days post egg-laying, the homozygous *Smn*^Δ/Δ^ larvae lacking “green balancers” were collected for the subsequent assays. Due to the weakness of the homozygous *Smn*^*Δ/Δ*^ larvae, here we carefully cultured all the larvae from the rescue crosses on agar plates instead of in typical vials to increase their chance of survival, and same for *WT* controls. The same strategy was used for rescue in the *U12*^Δ/ Δ^ background. All the other crosses were performed under standard conditions.

### Plasmid and cell culture

The CDS sequence of Dm65K was cloned into the modified pMT/V5-His B vector (Invitrogen) with hygromycin B and P copia promoter as described^69^. The construct was then transfected into *Drosophila* S2 cells by Effectene transfection reagent (Qiagen) and induced by 0.5 mM CuSO_4_ at 12 h after transfection. Cells were collected 24 h later for isolation of RNA and proteins.

### RNA-seq and RT-PCR

Total RNAs from larvae or S2 cells were isolated by TRIzol (Ambion) and treated with RNase-free DNase I (Invitrogen). Total RNAs from L3 wandering stage larvae of deletion and WT strains were subjected to paired-end mRNA sequencing by Illumina Hi-Seq 2000. For RT-PCR, cDNAs were reverse transcribed using RevertAid Reverse Transcriptase (Thermo) and amplified by Ex-Taq (TaKaRa).

### Western and northern blotting

Western blot signals of FLAG, tubulin, and SmD2 were detected using Monoclonal anti-FLAG M2-Peroxidase antibody (Sigma), mAb DM1A (Sigma) and anti-SNRPD2 antibody (Abcam), respectively. Northern analyses were performed by transferring RNAs from 8 M Urea-PAGE gel to Hybond-N membrane (Amersham) and probed by ^32^P-labeled antisense DNA oligonucleotides that are listed in Supplementary Data 5.

### NMJ and muscle size

Third-instar larvae at the wandering stage were dissected and stained as previously described^70^. Neurons were labeled and visualized by Alexa Fluor 488 conjugated goat anti-HRP IgG (Jackson Immuno Research) as described^71^, postsynaptic and presynaptic membrane were labeled by mouse anti Discs-Large antibody^72^ and visualized by Alexa Fluor 647 conjugated Rabbit Anti-Mouse IgG (Jackson Immuno Research). Types Ib and Is boutons at muscles 1 and 9 regions in abdominal segment A3 were identified as described^71,73^ and counted using a 60x oil objective on a Zeiss Axio Imager. Muscle 6 in abdominal segment A3 was visualized by its spontaneous fluorescence at 597 nm using a ×20 objective, muscle size was calculated by Photoshop CC (Adobe).

### Larva locomotion

Third-instar larvae at the wandering stage were individually placed on a 6-cm sucrose agar plate and their movement was recorded for 60 s by a digital camera at speed of 30 fps. To track the larvae path, a pencil tool with 3-pixels width was used for extracted and merged frames (1 out of 4) from video files by Photoshop (Adobe). The length of path was then calculated from the grayscale of pencil layer by imageJ (NIH), and normalized by the corresponding larva length, which is equal to the perimeter of half larva body based on the wrmTrck program (http://www.phage.dk/plugins/wrmtrck.html). For each strain, the five best performance larvae from eight tested were selected for data analyses.

### Hatching and survival rates

Parental strains were cultured in egg-laying cages with the sucrose agar-coated yeast paste for 2 h, and then the eggs were picked out and arranged on the agar plate 10 × 10 with keeping dosage appendix upward. After 36 h culture under 25 °C, hatched embryos were counted using a fluorescence microscope. For the investigation of survival rates, L1 larvae were cultured on a plate with no more than 20 individuals at 25 °C, and the survived Drosophila were counted every day.

### Co-immunoprecipitation

One gram of fly adults was ground with Buffer A (20 mM Hepes-KOH pH 7.9, 10 mM KCl, 1.5 mM MgCl_2_, 1% Triton X-100, 0.5% NP-40, 0.5 mM DTT, 1× cocktail protease inhibitor (Thermo), 0.5 U/μl recombinant RNA inhibitor (TAKARA) and 0.5 mM PMSF) in liquid nitrogen, and then homogenized in a Dounce homogenizer (Kimble). Cell pellets were then resuspended in Buffer B (600 mM KCl) and homogenized. Supernatants from the above two-steps were combined and KCl was adjusted to 100 mM before applying to ANTI-FLAG M2 Affinity Agarose Gel (Sigma). Co-purified proteins and RNA were further analyzed by western blotting and RT-PCR. Nuclear extract of S2 cells was prepared as described^74^ and applied to MBP-MS2-U11 ASO coated amylose resin (NEB) in Binding buffer (20 mM Hepes-KOH pH 7.9, 150 mM KCl, 0.2 mM sodium ortho-vanadate, 0.2 mM PMSF, 50 mM NaF, 2.5 mM MgCl_2_, 1× protease inhibitor (EDTA free), 0.25 U/μl recombinant RNase Inhibitor, 1% Triton X-100, 0.5% NP-40, 1.5 mM ATP and 5 mM creatine phosphate.

### Splicing analyses

Raw reads from RNA-seq were quality filtered and trimmed, and then mapped to the *Drosophila melanogaster* genome (dm6) by *TopHat*; changes of AS were analyzed by *rMATS*^75^.

To identify sensitive SSs in the mutant fly strains, we developed a bioinformatic tool to measure the Unused Index of each SS. First, all exon boundaries and SSs were collected according to the Flybase annotations and the newly identified SSs that were supported by >20 exon-exon junction reads in our deep sequencings. Second, as shown in Fig.2a, the unusage of each SS (SSun) was scored by the ratio of exonic boundary reads to intronic boundary reads. For example, the 5′SSun was scored by b1/a1, in which a1 is the number of all reads covering the last 40-nt of the upstream exon, and b1 is the number of all reads covering the first 40-nt of the intron. Likewise, the 3′SSun was scored by b2/a2. Third, comparison of each SSun between the mutant and WT strains were then performed through the measurement of Unused Index that was the product of the ratio (*R*) and difference (*D*) of SSun, $\frac{{kSS_{{\it{\mathrm{un}}}_{{\it{\mathrm{mutant}}}}}}}{{SS_{\mathrm{un}_{\mathrm{wt}}}}} \times (kSS_{{\it{\mathrm{un}}}_{{\it{\mathrm{mutant}}}}} - SS_{\mathrm{un}_{\mathrm{wt}}})$, *k* is the reads normalization between two strains. Rather than using a single *R* or *D* value, the product of *R* and *D* was used to reduce the potential inaccuracy of *R* generated from small values of SSun and *D* generated from large values of SSun. Finally, the top 2% SSs with high ranking of Unused Indexes (around 2.5–30.0) from each mutant strain were selected for further analyses (Fig. 2b).

To detect changes of the single minS-SS-related AS, *PSI* of its alternative SS involved splicing event was calculated by formula *J*_alternative_/(*J*_alternative_ + *J*_sensitive_) in each sample, and was then normalized by Z-score.

### Free energy analysis

Sequences of 25 nts (positions from −5 to +20) at the 5′SS regions and 20 nts (position from 1 to 20) of the U11 snRNA were selected for measuring stabilities (free energies) of the 5′SS:U11 RNA duplexes using *RNAduplex* (version 2.4.3)^52,76^. As the identified single minS-3′SSs, sequences of their corresponding 5′SSs were selected based on junction reads in our RNA-seq.

### Reporting summary

Further information on research design is available in the Nature Research Reporting Summary linked to this article.

## Supplementary information

Supplementary Information

Description of Additional Supplementary Files

Supplementary Data 1

Supplementary Data 2

Supplementary Data 3

Supplementary Data 4

Supplementary Data 5

Reporting Summary

## Data Availability

Next-generation sequencing has been submitted to the Gene Expression Omnibus (accession number GSE138183). Source data are provided with this paper.

## Source data

Source Data

## Code availability

Codes of Unused Index are available from github (https://github.com/Xu-Lab-WHU/UnusedIndex). All other data will be made available on request.
