# Supplementary material for: Defective minor spliceosomes induce SMA-associated phenotypes through sensitive intron-containing neural genes in Drosophila
Source: Nat Commun. 2020 Nov 5;11:5608. doi: 10.1038/s41467-020-19451-z (PMC7644725; doi:10.1038/s41467-020-19451-z)
Supplement: Supplementary file 1 — Supplementary Information [file 41467_2020_19451_MOESM1_ESM.pdf]

Supplementary information

Includes eight supplementary figures.

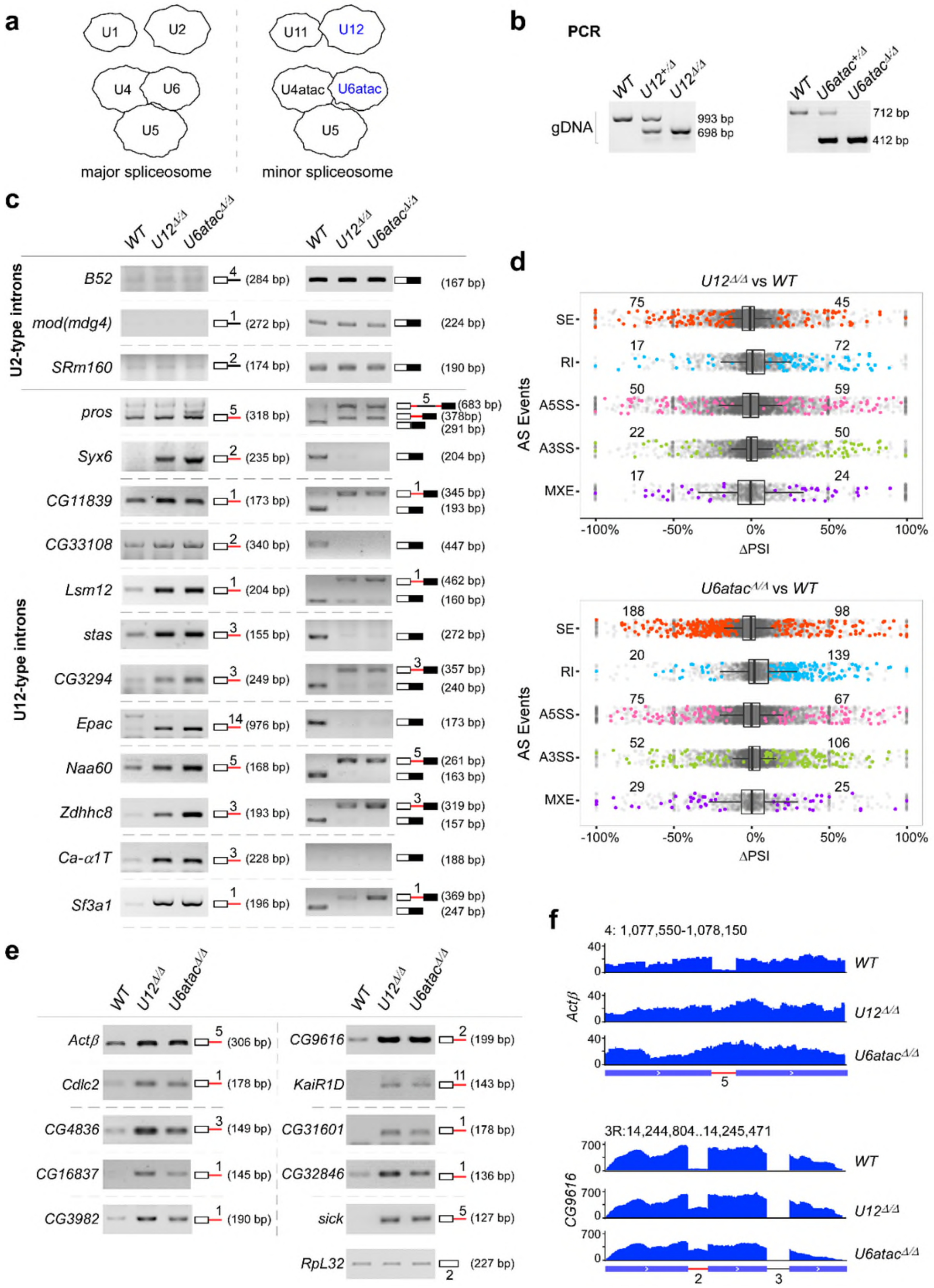

Supplementary Fig. 1: U12-type Introns are retained in the *U12*$^{\Delta/\Delta}$ and *U6atac*$^{\Delta/\Delta}$ strains.

a, Diagram of snRNAs in the major and minor spliceosomes. **b**, Validation of the deletion strains by amplification of genomic DNAs. Both heterozygotes and homozygotes are tested. **c**, Splicing of all known U12-type introns are inhibited in the two deletion strains. Red lines: U12-type introns; black

lines: U2-type introns. Amplification of *RpL32*-exon 2 was used as loading control. **d**, Transcriptome-wide analyses of alternative splicing changes by rMATs. Five types of AS events are indicated. n=6329 (SE); n=2221 (RI); n=7318 (A5SS); n=5694 (A3SS) and n=1419 (MXE). **e**, RT-PCR validation of the newly identified minS-Is that are retained in both deletion strains. **f**, Examples of new minSIs with distribution of RNA-seq reads in *Drosophila* strains. minS-Is are indicated in red. In **d** boxplots, the middle line show data median, the lower and upper hinges correspond to the 25th and 75th percentiles, the upper whisker extends from the hinge to the largest value no further than 1.5 × IQR from the hinge (where IQR is the interquartile range) and the lower whisker extends from the hinge to the smallest value at most 1.5 × IQR of the hinge. Source data are provided as a Source Data file.

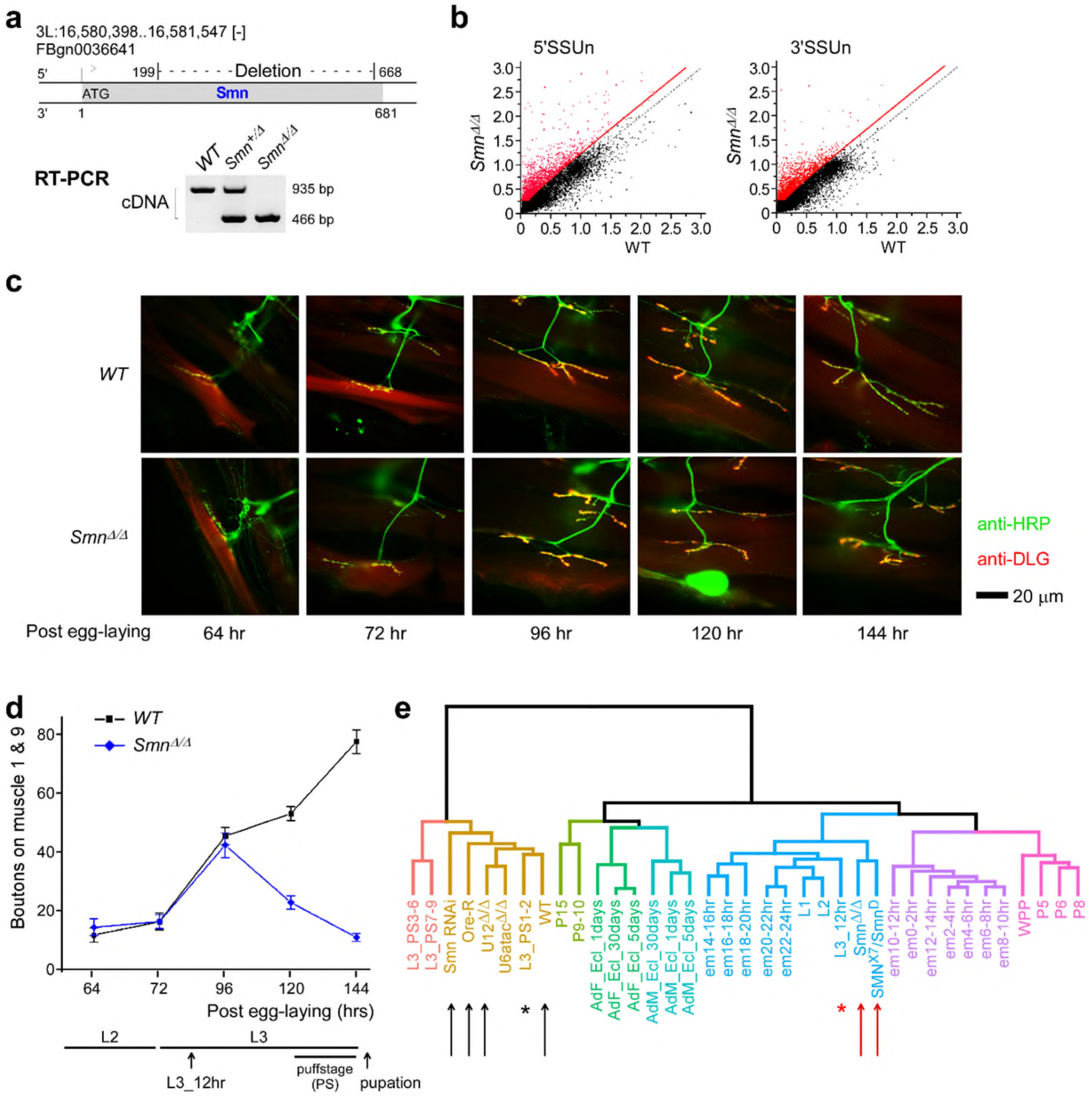

Supplementary Fig. 2: The *Smn*$^{\Delta/\Delta}$ strain exhibits stronger SMA-associated phenotypes

a, Construction of the *Smn*$^{\Delta/\Delta}$ strain by CRISPR/Cas9 system. Both heterozygotic and homozygotic deletion strains are validated by PCR and sequencing. **b**, Distribution of SSun scores of transcriptome-wide 5'SSs and 3'SSs in the *Smn*$^{\Delta/\Delta}$ strain versus the *WT* strain. Grouped by a red unbroken limitation line, red dots represent the top 5% of SSun scores in the mutant strain; and black dots represent the remaining SSun scores. **c**, Degeneration of motor neurons occurred in the *Smn*$^{\Delta/\Delta}$ strain in the middle of L3 stage. Green: neuron visualized by HRP antibody; red: presynaptic and posterior membranes visualized by DLG antibody. Boutons of NMJ in the colocalized regions are

counted under microscopy. **d**, Quantitation of the NMJ Boutons during the time course (n=3 for each group). **e**, The sequenced *Smn*$^{\Delta/\Delta}$ strain is at the stage after L3_12hr. Transcriptomic data, including the modEncode Drosophila data sets (Graveley, 2011), *Smn* mutant (Garcia, 2003) and other strains used in this study, are analyzed by the software factoextra R package. Data are presented as mean values +/- SD in **d.** Source data are provided as a Source Data file.

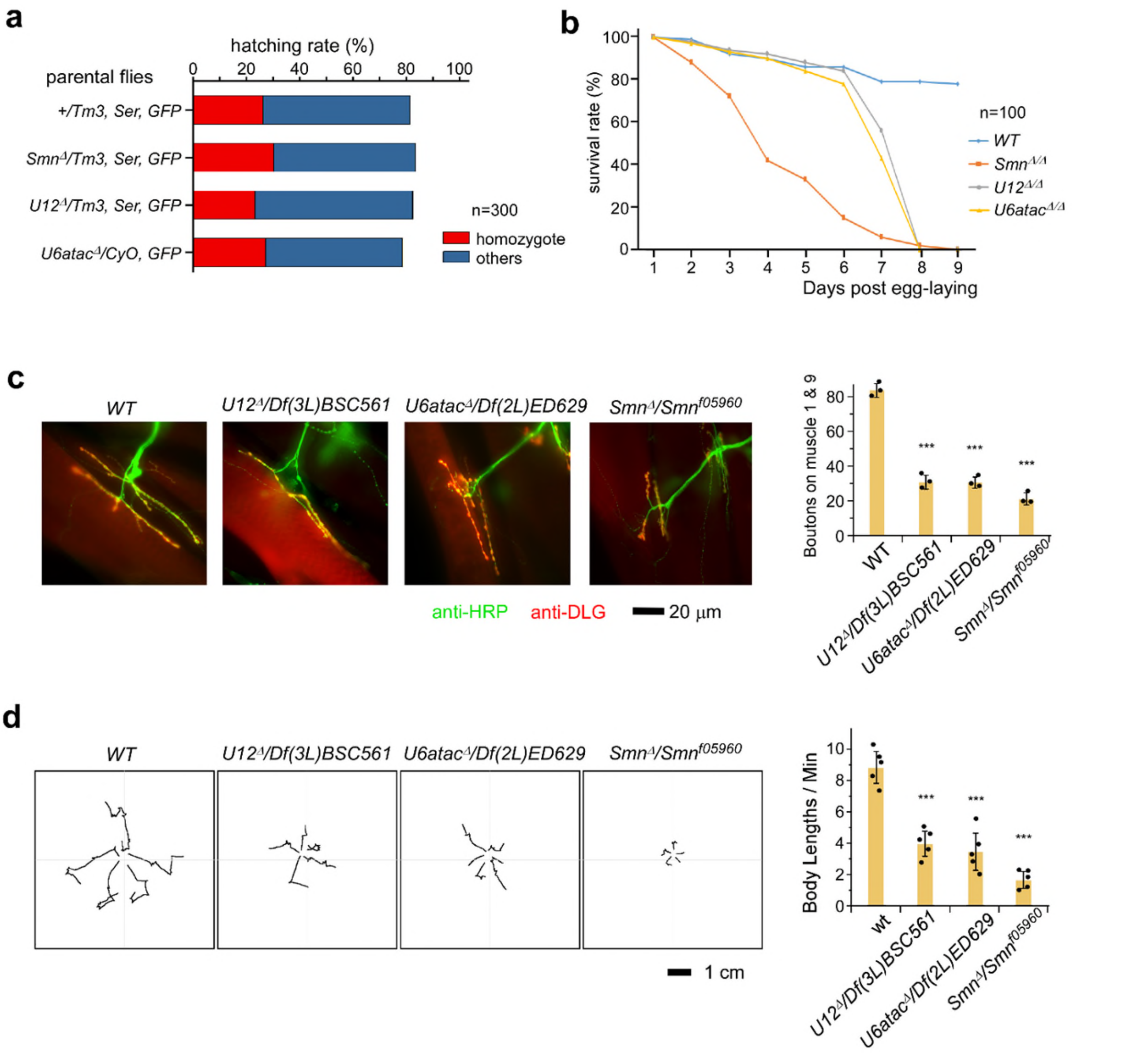

Supplementary Fig. 3: Characterizations of the CRISPR-Cas9 generated *U12*$^{Δ/Δ}$, *U6atac*$^{Δ/Δ}$ and *Smn*$^{Δ/Δ}$ strains.

a, Hatching rates of the homozygotic deletion strains (*Smn*$^{Δ/Δ}$, *U12*$^{Δ/Δ}$ and *U6atac*$^{Δ/Δ}$) are similar as the *WT* strain. **b**, Survival rates of the *Smn*$^{Δ/Δ}$, *U12*$^{Δ/Δ}$ and *U6atac*$^{Δ/Δ}$ strains. Cross-over of the deletion strains with either deficiency strain or P-element insertion mutant strain exhibits similar SMA-associated phenotypes, including the boutons numbers of NMJ (**c**) (n=3 for each group, *U12*^{Δ}/ *Df(3L)BSC561*: p = 8.8e-5; *U6atac*^{Δ}/*Df(2L)ED629*: p = 8.2e-5; *Smn*^{Δ}/ *Smn*^{f05960}: p = 4.1e-5) and larval locomotion (**d**) (n=5 for each group, *U12*^{Δ}/ *Df(3L)BSC561*: p = 9.1e-5; *U6atac*^{Δ}/*Df(2L)ED629*: p = 0.00014; *Smn*^{Δ}/ *Smn*^{f05960}: p = 1.5e-5). The deficiency allele *Df(3L)BSC561* (Lee et al., 2018) is used for *U12*$^{Δ/Δ}$, *Df(2L)ED629* (Ryder et al., 2007) for *U6atac*$^{Δ/Δ}$,

and *Smn*^{f05960} (Chang et al., 2008) allele *Smn*$^{\Delta/\Delta}$. Data are presented as mean values +/- SD in **c** and **d,** ***p < 0.001. *P* values were calculated using two-sided *t*-test. Source data are provided as a Source Data file.

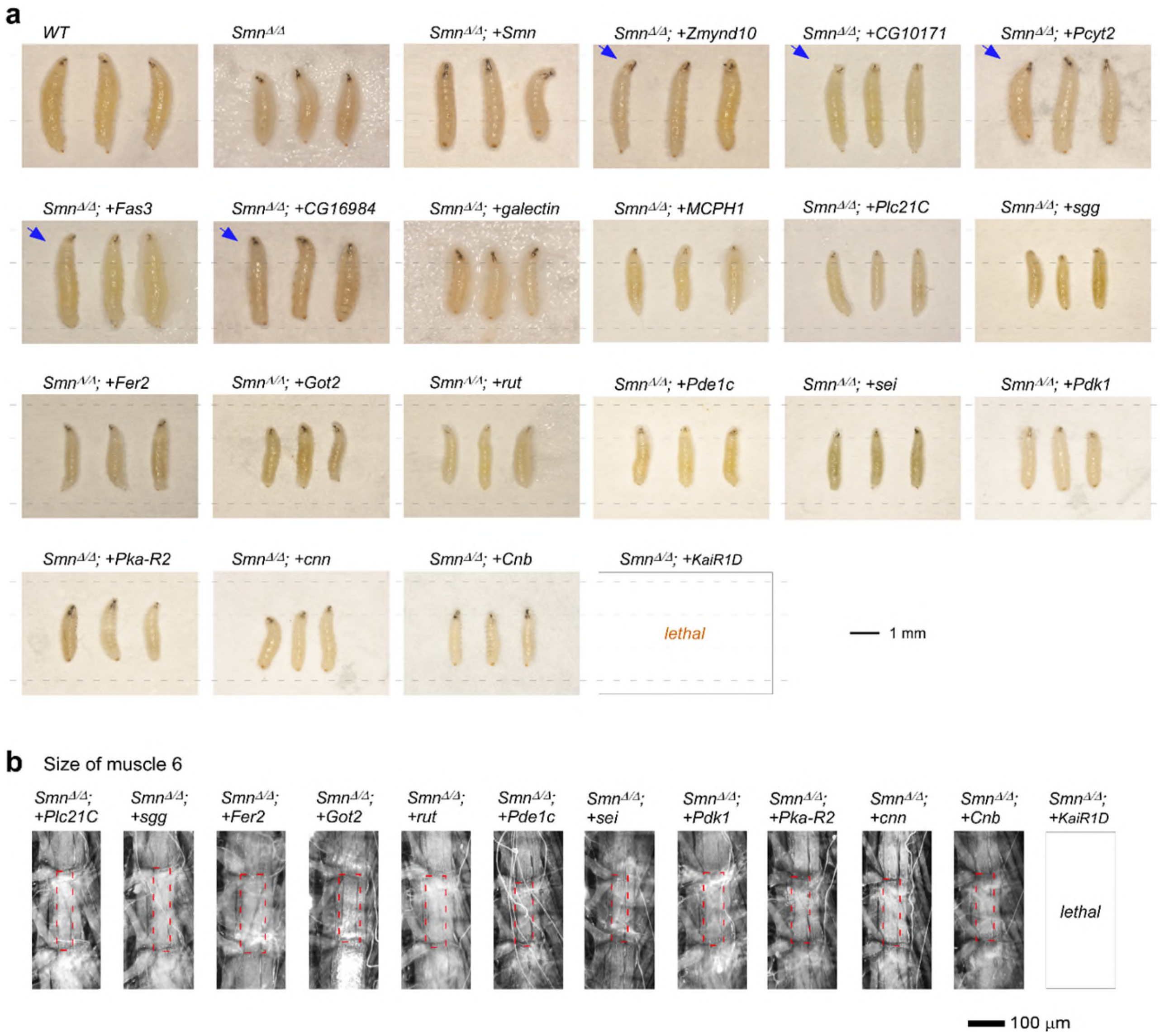

Supplementary Fig. 4: Body size and muscle development of L3 larvae in the *Smn*$^{\Delta/\Delta}$ background.

a, Larvae body size in the *Smn*$^{\Delta/\Delta}$ background is restored by expression of CDS of five genes (blue arrows) driven by *elav-GAL4*. **b**, Muscle 6 of twelve non-rescuing transgenic strains are presented, others are shown in Fig. 2e.

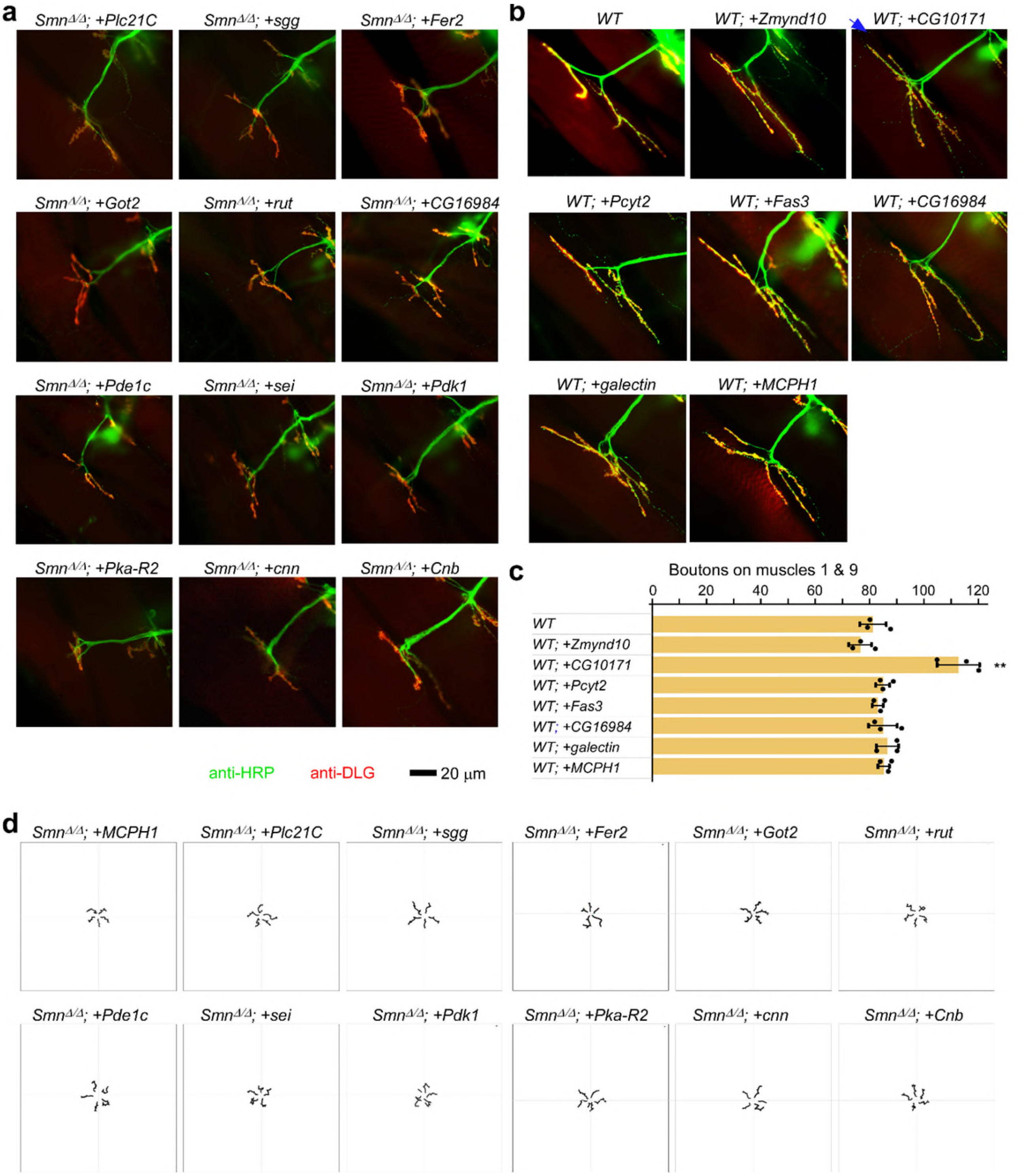

Supplementary Fig. 5: Detection of NMJ and larvae mobility of transgenic strains.

a, Immunostaining of neuromuscular connections of twelve negative CDSs strains in the *Smn*$^{\Delta/\Delta}$ background. Quantitation of NMJ boutons are shown in Figure 6a. **b**, Immunostaining of neuromuscular connections of transgenic strains in the *WT* background. **c**, Quantitation of NMJ boutons of transgenic strains in the *WT* background. Expression of *CG10701*-CDS significantly changes NMJ (*WT*; *+CG10171*: p = 0.00686). Data represent the mean ±SD from five

representatives of each strain, **$p < 0.01$. **d**, Locomotion of twelve negative transgenic strains. Quantitation of larval path lengths is shown in Fig. 6b. *P* values were calculated using two-sided *t*-test. Source data are provided as a Source Data file.

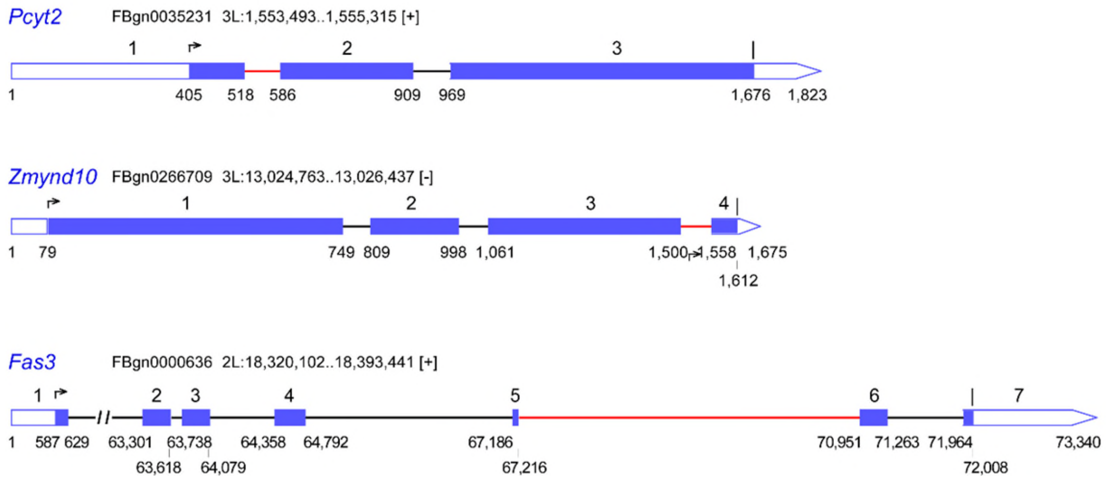

Supplementary Fig. 6: Schematic of the three neural genes that rescue SMA-associated phenotypes in *Drosophila.*

Empty boxes, UTRs; blue boxes, CDSs; Line, introns; start and stop codons are indicated. Retained introns are marked in red.

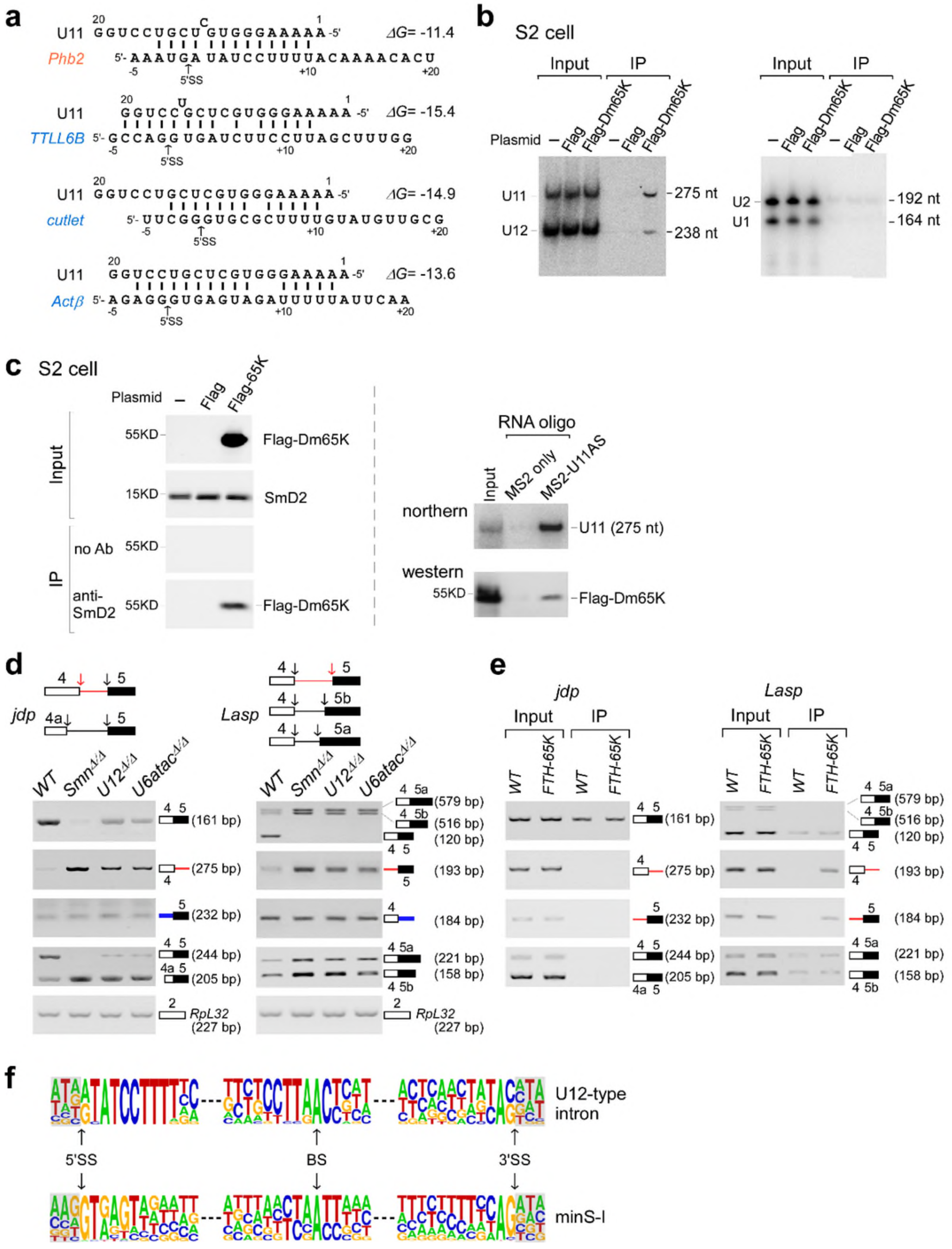

Supplementary Fig. 7: Dm65K protein is a specific U11/U12 di-snRNP component in *Drosophila* and Co-IPs minS intron-containing pre-mRNA.

a, Examples of stable 5'SS:U11 RNA duplexes. Orange: U12-type intron; blue: new minS-I. Base-pairs and free energy are indicated. **b**, Flag-Dm65K, expressed in S2 cells, specifically co-purifies U11 and U12 snRNAs that are visualized by northern blot. **c**, Flag-Dm65K is co-purified by

spliceosomal components in S2 cells. Left, SmD2 antibody co-purifies Dm65K; Right, MS2 linked anti-sense oligo of U11 snRNA pulls down Dm65K. **d**, The common SSs can be recognized by both the major and minor spliceosomes. Four sets of primers were used to amplify various pre-mRNAs and mRNAs. **e**, Pre-mRNAs, but not mRNAs, of the minS intron-containing genes are enriched by Dm65K. FTH-Dm65K and the *WT* strains were used. **f**, Intronic consensus sequences of the newly identified minS-Is. Source data are provided as a Source Data file.

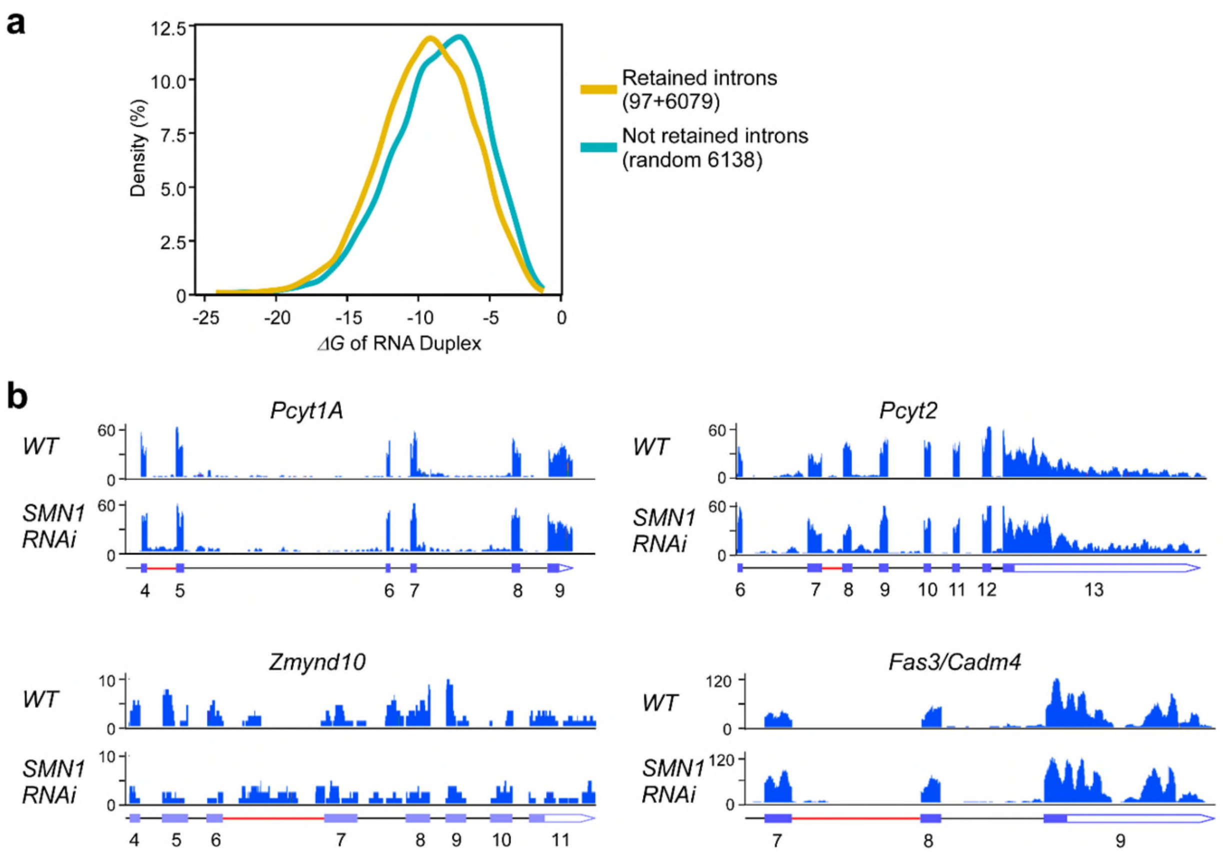

Supplementary Fig. 8: Target genes of *Smn* and effectors of SMA-associated phenotypes are conserved across species.

a, Density distribution of the 5'SS:U11 RNA duplex stabilities. Retained and not retained introns were identified from a published sequencing dataset from SMN1 knock-down human SH-SY5Y cells (Jangi et al., 2017). **b**, Retained introns (red) of human homologs of *Drosophila Zmynd10*, *Pcyt2*, and *Fas3* in SMN1 knock-down SH-SY5Y cells.
