# Supplementary material for: Defective minor spliceosomes induce SMA-associated phenotypes through sensitive intron-containing neural genes in Drosophila
Source: Nat Commun. 2020 Nov 5;11:5608. doi: 10.1038/s41467-020-19451-z (PMC7644725; doi:10.1038/s41467-020-19451-z)
Supplement: Supplementary file 2 — Description of Additional Supplementary Files [file 41467_2020_19451_MOESM2_ESM.pdf]

Description of Additional Supplementary Files

Supplementary Data 1: Mapping results of RNA-seq.

Supplementary Data 2: Unused index of splice sites in the deletion strains.

Supplementary Data 3: Introns that are sensitive to defective minor spliceosomes.

Supplementary Data 4: Candidate genes and their minor spliceosome-sensitive splice sites.

Supplementary Data 5: List of primers and oligos used in this study.
