# Supplementary material for: Defective minor spliceosomes induce SMA-associated phenotypes through sensitive intron-containing neural genes in Drosophila
Source: Nat Commun. 2020 Nov 5;11:5608. doi: 10.1038/s41467-020-19451-z (PMC7644725; doi:10.1038/s41467-020-19451-z)
Supplement: Supplementary file 3 — Supplementary Data 1 [file 41467_2020_19451_MOESM3_ESM.docx]

**Li _** **Supplementary Data 1**

**Supplementary Data 1. Mapping Results of RNA-seq in Fruit Fly Samples**

| **Sample** | **Total reads** | **Mapped reads** | **Mapping ratio** |
| --- | --- | --- | --- |
|  |  |  |  |
| WT (5905)_1 | 67,487,514 | 64,024,353 | 94.87 |
| WT (5905)_2 | 60,587,504 | 58,727,485 | 96.93 |
| *U12^Δ/Δ^* _1 | 67,680,456 | 61,861,869 | 91.40 |
| *U12^Δ/Δ^*_2 | 60,750,924 | 58,038,945 | 95.54 |
| *U6atac^Δ/Δ^*_1 | 61,968,502 | 58,623,872 | 94.60 |
| *U6atac^Δ/Δ^*_2 | 60,225,672 | 55,920,262 | 92.85 |
| *Smn^Δ/Δ^* _1 | 56,195,760 | 51,243,516 | 91.19 |
| *Smn^Δ/Δ^* _2 | 50,266,710 | 46,375,312 | 92.26 |
