# Supplementary material for: Defective minor spliceosomes induce SMA-associated phenotypes through sensitive intron-containing neural genes in Drosophila
Source: Nat Commun. 2020 Nov 5;11:5608. doi: 10.1038/s41467-020-19451-z (PMC7644725; doi:10.1038/s41467-020-19451-z)
Supplement: Supplementary file 7 — Supplementary Data 5 [file 41467_2020_19451_MOESM7_ESM.docx]

**Li_** **Supplementary Data 5**

**Supplementary Table 5. List of Primers and Oligos Used in This Study**

| **Primers** | **Sequences** | **Notes** |
| --- | --- | --- |
| LL001 | 5'_AAGGGGGGATCTAGATCGGGATGAGCGCGCTTAGCAGTAAAC | Dm65K F primer homo to pMT (Kpn I) |
| LL002 | 5'_TCATGGTCTTTGTAGTCAGCTTGCTGTTTGCCGTAGCAG | Dm65K R primer homo to pMT (Not I) |
| LL003 | 5'_CATTGGTCAGATCCTCAC | dU12 genome F primer |
| LL004 | 5'_GACAACAACTGATTCAGG | dU12 genome R primer |
| LL005 | 5'_TCAGTGTCAATGTTAGCCAG | dU6atac genome F primer |
| LL006 | 5'_GAATCTCTAGTGCGTAGTG | dU6atac genome R primer |
| LL007 | 5'_CCGCATATGGTCCACCAATC | Dm65K N-ter knock-in primer 1 F |
| LL008 | 5'_TTCCTTGTCATCGTCATCCTTGTAATCGATATCATGATCTTTATAATCACCGTCATGGTCTTTGTAGTCCATTTTCGTCACAATTTCCAGCGAGC | Dm65K N-ter knock-in primer2 tag R |
| LL009 | 5'_GGATGACGATGACAAGGAAAATTTATATTTTCAAGGTCATCATCACCATCACCATATGAGCGCGCTTAGCAGTAAACTTCTCATGAAACGGATGCCCTGCTCGtTaGACGAACTGGCGCAGCTGATCC | Dm65K N-ter knock-in primer3 tag mut F |
| LL010 | 5'_CTCGAGGAGCACCCTTTTCCGGCCGTAGGTGTTCAGTGAcctgGGCAGAATGAGCTTGCACTCACGGATCAGCTGCGCCAGTTCGTC | Dm65K N-ter knock-in primer4 mut R |
| LL011 | 5'_CCGGAAAAGGGTGCTCCTCGAGT | Dm65K N-ter knock-in primer5 F |
| LL012 | 5'_CGCAACACTGAAATCCGACT | Dm65K CDS F (NTAP screen F) |
| LL013 | 5'_TCGCTGAGTTCTTGGAGCTG | Dm65K CDS R (NTAP screen R) |
| LL014 | 5'_TAGGATCATGGGACGCAAGA | BuGZ ex1 F |
| LL015 | 5'_CGGCGTTGTAGCCAGTATGC | BuGZ in1 R |
| LL016 | 5'_GTCCACCGTTTCCTTGTGCA | BuGZ ex2 R |
| LL017 | 5'_TTCCCCGAGATCTCTATCAG | Ca-α1T ex4 F |
| LL018 | 5'_TCAATCCACCAAACGAACAC | Ca-α1T in4 R |
| LL019 | 5'_CAGTTGAGCAGGATGACCAG | Ca-α1T ex5 R |
| LL020 | 5'_GAGGCCAAACAAGTGGCTCAGCAGGA | Phb2 ex4 F |
| LL021 | 5'_CCAGCTCACAACATACTAAC | Phb2 in4 R |
| LL022 | 5'_CGTGCGTGCAATGCTTTGAG | Phb2 ex5 R |
| LL023 | 5'_GATCAGCCCAAGTCGATGTC | Sf3a1 ex1 F |
| LL024 | 5'_GACATCGACTTGGGCTGATC | Sf3a1 in1 R |
| LL025 | 5'_TCCGGTCCATTGCGAGCCAC | Sf3a1 ex2 R |
| LL026 | 5'_GCATGTTGCTTGGTGGCAGT | CG33108 ex2 F |
| LL027 | 5'_CACTGCCAGACAACAGTGTG | CG33108 in2 R |
| LL028 | 5'_GCATGGTTTACATCGGCATCGT | CG33108 ex3 R |
| LL029 | 5'_CACTCATTCCTGCCCTACTAC | Naa60 ex5 F |
| LL030 | 5'_GTATAGCGAAATCATAGAGAG | Naa60 in5 R |
| LL031 | 5'_GCACCATCGACGCGTAGTGC | Naa60 ex6 R |
| LL032 | 5'_GATGCTAAGATGCTAGCCAAG | stas ex3 F |
| LL033 | 5'_GCGACCGACTTTTCATGCCA | stas in3 R |
| LL034 | 5'_AGCAGAATCGAGAGGAACAG | stas ex4 R |
| LL035 | 5'_CAGCAGTCACTGTGGATTCA | CG11839 ex1 F |
| LL036 | 5'_GTTCAGTTAAGGAAACCAACTAGC | CG11839 in1 R |
| LL037 | 5'_GGATCCTTGGGTGCAGTAGT | CG11839 ex2 R |
| LL038 | 5'_GAGATGCTAGCCTCCGACCA | Kcmf1 ex3 F |
| LL039 | 5'_GCCTTTTGTATGGATGCAGT | Kcmf1 in3 R |
| LL040 | 5'_GAACTGAAGTGCATGTTGGA | Kcmf1 ex4 R |
| LL041 | 5'_CAGACAGACCACAACGAGCA | Ca-α1D ex5 F |
| LL042 | 5'_CCAGATTTAGGCTTCACATC | Ca-α1D in5 R |
| LL043 | 5'_CAAGGTTTGATTCGTCACGTTTG | Ca-α1D ex6 R |
| LL044 | 5'_GGATTCCTGCTGATTGGAGTG | Tsp97E ex2 F |
| LL045 | 5'_GACCACGACTGTAGCTGCCT | Tsp97E in2 R |
| LL046 | 5'_GATGGAGAACTGGATCAGGA | Tsp97E ex3 R |
| LL047 | 5'_GAGGCTGAATGGACCACCAC | Syx6 ex2 F |
| LL048 | 5'_CTGATGAGCTAGAAAGTCGGT | Syx6 in2 R |
| LL049 | 5'_GTGTGCGGTGATGTCTCTGT | Syx6 ex3 R |
| LL050 | 5'_TCTGCACTTCGCGCATTTACCAATC | CG3294 ex3 F |
| LL051 | 5'_CTTTGGGACATTTACGTGTTAAGG | CG3294 in3b R |
| LL052 | 5'_ACAGGTTGTTCGGATTGCCA | CG3294 ex4 R |
| LL053 | 5'_GCGATTTCTTCTCCATCTTC | Nhe3 ex5 F |
| LL054 | 5'_GGAAGTCATTAGCATAGGTGT | Nhe3 in5 R |
| LL055 | 5'_CACACCAGTAAGTTCCGTAG | Nhe3 ex6 R |
| LL056 | 5'_CATCGGATCCACAGTCGTCT | Lsm12a ex1 F |
| LL057 | 5'_GCTCAAGAGATCCCTGTGGA | Lsm12a in1 R |
| LL058 | 5'_GCTGCAGAGCGAGAGGTTCA | Lsm12a ex2 R |
| LL059 | 5'_CATCAGCACCACCAGCAGCA | pros ex5 F |
| LL060 | 5'_CAGCGACTCCTGGTGTTCTGT | pros in5a R |
| LL061 | 5'_GTGCATCGGTGTCAATGTAGA | pros ex6 R |
| LL062 | 5'_GCTGAGCATCTTCTCGCTGT | Zdhhc8 ex5 F |
| LL063 | 5'_GCAGATGCCTACGCTCCCTT | Zdhhc8 in5 R |
| LL064 | 5'_CGACACCAGTACCATGTGGA | Zdhhc8 ex6 R |
| LL065 | 5'_CTTGGACGACTTCCTGCTCA | Epac ex14 F |
| LL066 | 5'_GGACCTAGCTAAACGGACCT | Epac in14 R |
| LL067 | 5'_GCCATGACCCACTTTTGCATG | Epac ex15 R |
| LL068 | 5'_CTCTGGACATTCTACAGCCA | nmo ex5 F |
| LL069 | 5'_GTTCCGAGTTCATCAAAGGGTAGA | nmo in5 R |
| LL070 | 5'_GATCTGGTACAGGAACACCT | nmo ex6 R |
| LL071 | 5'_GCTTTGGCAGCCCGTTGACA | CG33108 specific RT primer |
| LL072 | 5'_ACTTGCGCGAGGAGTTGCTG | eIF4a ex2 F |
| LL073 | 5'_TGGATCTGCGTGGCCAACTC | eIF4a ex3 R |
| LL074 | 5'_GATGATGGTGAAGATGATGATG | eIF4a in2 R |
| LL075 | 5'_CAGATGACGTTATCGTTGGTGT | βTub60D ex1 F |
| LL076 | 5'_CATGCTCATCGGAGATGATCTC | βTub60D ex2 R |
| LL077 | 5'_TTCTTAAGACTGAGTTAGAAAAC | βTub60D in1 F |
| LL008 | 5'_AATGGATGCCGACAGCAGTG | tra ex1 F |
| LL079 | 5'_CAGATTCTGATCCAGTGCCAT | tra int1 F |
| LL080 | 5'_TGTGCGGTTAGTCAATGCATC | tra ex2 R |
| LL081 | 5'_GCAAGCCCAAGGGTATCGACA | Rpl32 ex2 F |
| LL082 | 5'_AATCTCCTTGCGCTTCTTGGAGGAG | Rpl32 ex2 R |
| LL083 | 5'_GCGCTTTCCAACACACAGCA | DmSmn genome F |
| LL084 | 5'_CGAAAAGATGTGATGTGATGATG | DmSmn genome R |
| LL085 | 5'_GCACTAGGTTGGGATGACTGGAT | Actβ ex5 F |
| LL086 | 5'_AGGTAAAATAAGATTGTGTTATTAC | Actβ in5 R |
| LL087 | 5'_CGATGTCCTTCTCGATGTTG | Cdlc2 in1 F |
| LL088 | 5'_CACAGGTAGCCCCAAAGCCT | Cdlc2 ex2 R |
| LL089 | 5'_TGCTTCAGCGACGAAGACAG | CG4836 ex3 F |
| LL090 | 5'_GACCTGAGGGAACTGAGCAA | CG4836 in3 R |
| LL091 | 5'_ATGTCCAGAAGTTCGAGGTT | CG16837 ex1 F |
| LL092 | 5'_CTAGGGAGATGGACAAATGC | CG16837 in1 R |
| LL093 | 5'_TCCTCGCGATTTGTGCTGAA | CG3982 ex1 F |
| LL094 | 5'_CAGTCATCATCTCGAGGAGT | CG3982 in1 R |
| LL095 | 5'_CAGCACTACTGCGATGAGCA | CG9616 ex2 F |
| LL096 | 5'_GGGAAGTTCAAAGATGCAGA | CG9616 in2 R |
| LL097 | 5'_GATCTCCACATATCAGCGCA | KaiR1D ex11 F |
| LL098 | 5'_TTGCGTATTCCTTTCTCTCAA | KaiR1D in11 R |
| LL009 | 5'_GAAGGTGAGAAGATTTTTGGGAA | CG31601 in1 F |
| LL100 | 5'_CATCATCTCCTTCAAGTTGT | CG31601 ex2 R |
| LL101 | 5'_GAACCCAATGGTTGCTGAAG | CG32846 ex1 F |
| LL102 | 5'_GAACTGCTGGTACATCGTATC | CG32846 in1 R |
| LL103 | 5'_GGACTTGGAAAGAGCGGAAG | sick ex5 F |
| LL104 | 5'_ACGTGATGACCTCAAAAGAATCT | sick in5 R |
| LL105 | 5'_CTCTCCAAGACGGTAAGTTG | SRm160 in2 F |
| LL106 | 5'_GCTGCCACATCTGCCGATGT | SRm160 ex3 R |
| LL107 | 5'_ACTATTGTTGCTTTTATTTGCACG | B52 in4 F |
| LL108 | 5'_TAACGCGGCTAGACAAATTCTCC | B52 ex5 R |
| LL109 | 5'_TACTTACCTGGCGTAGAGG | U1 F |
| LL110 | 5'_TCGGGACGGCGCGAACGCC | U1 R |
| LL111 | 5'_CGTTTCCGATCACGAAAC | U11 F |
| LL112 | 5'_CGCAGGGAGATCCGGGAAT | U11 R |
| LL113 | 5'_CTAATGAGTAAGGAAAAC | U12 F |
| LL114 | 5'_AAGTAGGCGCCGGTTCGCT | U12 R |
| LL115 | 5'_GTTGTGGAGAACTAGGTGCA | HipHop in1 F |
| LL116 | 5'_CGGTAGAGTCGCACCAGGTC | HipHop ex2 R |
| LL117 | 5'_ACGACCACCAAGAAATTGGT | CG34161 ex1 F |
| LL118 | 5'_GTGATGTATTTAATCAGCTAAT | CG34161 in1 R |
| LL119 | 5'_CAATCCCAAGTACGGCGAAA | CG4270 ex2 F |
| LL120 | 5'_GGAAGACATGTATGAATAGG | CG4270 in2 R |
| LL121 | 5'_GACGCATTAGTTGTCCCAGA | tea ex8 F |
| LL122 | 5'_GTATAAATGTGCTTGCTAATCC | tea in8 R |
| LL123 | 5'_ACGTTCGACGATCAAAGATG | Fer1HCH ex1 F |
| LL124 | 5'_CTGGTTTGGTAACTTCACTTC | Fer1HCH in1 R |
| LL125 | 5'_GGAGCCGCTGAAAGCCAAAG | CG31068 ex1 F |
| LL126 | 5'_ACTCGAGCAGTTAGATTTAGTTG | CG31068 in1 R |
| LL127 | 5'_GCAATGGACAGAATGCCGAA | Pcyt2 ex1 F |
| LL128 | 5'_GTGGCAACTCCTGTTCCTCT | Pcyt2 in1 R |
| LL129 | 5'_GTTGCCACATACTAATCTCT | Pcyt2 in1 F |
| LL130 | 5'_CGTCGTCTTCCCAGCTCTGG | Pcyt2 ex2 R |
| LL131 | 5'_GCCTACGGCACGGACTTGCT | Zmynd10 ex3 F |
| LL132 | 5'_GAAGCGAACTCCGCAAAAGT | Zmynd10 in3 R |
| LL133 | 5'_TGGACGCAACTTTTGCGGAG | Zmynd10 in3 F |
| LL134 | 5'_CTAGACGCTGGGTCACAATTAAG | Zmynd10 ex4 R |
| LL135 | 5'_CAAGACGCCCACAAACGAAAC | Fas3 ex5 F |
| LL136 | 5'_CCAGATACCGTCGACGGGTT | Fas3 in5 R |
| LL137 | 5'_CCACGGATCCACAACTAACC | Fas3 in5 F |
| LL138 | 5'_CAACCGACAACCGATCCTGCT | Fas3 ex6 R |
| LL139 | 5'_CTACTACTTTCATATGGACGACA | Fas3 in5a F |
| LL140 | 5'_CCGATCCTGCTGGCCATTTCC | Fas3 ex6a R |
| LL141 | 5'_GTCTAGTCTCAAGTGCACCA | Taf4 ex3 F |
| LL142 | 5'_GATTGGGGATCAGATTTGGACGA | Taf4 in3 R |
| LL143 | 5'_CTGCTGCATGATGTAGGCTA | Taf4 in3 F |
| LL144 | 5'_CCTGTTGGCGCATGCCCACCA | Taf4 ex4 R |
| LL145 | 5'_GGTCAGCATCAGGGACTTCAG | Taf4 ex3a F |
| LL146 | 5'_GCTGGTGCACTTGAGACTAGAC | Taf4 in3a R |
| LL147 | 5'_GTGAGAAGATACACGATTGCCGA | jdp ex4 F |
| LL148 | 5'_CCGAAGTGAGGAAGTGAGAGGGCA | jdp in4 R |
| LL149 | 5'_CACCCACTCACGCTACGCCAACCA | jdp in4 F |
| LL150 | 5'- CTGGGCAGCTGCTCCACCGGTCTC | jdp ex5 R |
| LL151 | 5'_GAGGGCCATCTACGACAAGTGG | jdp ex4a F |
| LL152 | 5'_CTCTTTATCCACTTTCTCGGCAATCGTG | jdp in4a R |
| LL153 | 5'_CACGAAGCACATATCGAATGTG | Lasp ex4 F |
| LL154 | 5'_CAGTCCTCTCACAGAGATGGA | Lasp in4 R |
| LL155 | 5'_GCACACTACCAACAACCGCA | Lasp in4 F |
| LL156 | 5'_GCGCATCTCGTTGTAGCTGTTG | Lasp ex5 R |
| LL157 | 5'_GCAGTTGCTGCTGATGTTGCT | Lasp ex5b R |
| LL158 | 5'_GTGCTTTCTGAACTACCGTTAACC | Lasp in4a F |
| LL159 | 5'_GGTGCATATTGCGAGAATTGTTCAGCT | Lasp ex5a R |
| LL160 | 5'_CACCCAGATCGCGTTGAGCGA | Pyd ex5 F |
| LL161 | 5'_CTACATTGGACAATACTCAGACAAC | Pyd in5 R |
| LL162 | 5'_GCCAAACAATTGCGCTCAATCC | Pyd in5 F |
| LL163 | 5'_GACTCAGTTCCAAATCGGATTCG | Pyd ex6 R |
| LL164 | 5'_GTAGCAGCTGCTGCTGTGTCAGCTGA | Pyd ex6a R |
| LL165 | 5'_CTTACAAACATGCCCATGATTGTTGTCAACG | Pyd in5a F |
|  |  |  |
| LL166 | 5'_TTATTCACATTAGGAATAATCGCAGAGGTCAACTCAGCCGAGGTGCAATGGCCAAGCCTC | Dm U1 probe 60 nt |
| LL167 | 5'_CCCGTGACAGAGGTGGAGCAAGCCCCTAGCACTCCGTCTGATTCCAAAAATCAGTTTAAC | Dm U2 probe 60 nt |
| LL168 | 5'_ACGCTCCTGCGGCGCAATGCGTCCGCACTTGAGTTTCGTGATCGGAAACGTGCCAGGACG | Dm U11 probe 60 nt |
| LL169 | 5'_GACCTGTGCCCTTGCAAAAATTTGAGCTTCAAGGGGCGCGGGACACGCCCCCCTGCCTAG | Dm U12 probe 60 nt |
| LL170 | 5'_CGTAGTGGACGGTATTTCACGTCCCCATGGCGGGGTATTGGTTAAAGTTTTCAACTAGC | Dm U4 probe 59 nt |
| LL171 | 5'_TCACGATTTTGCGTGTCATCCTTGCGCAGGGGCCATGCTAATCTTCTCTGTATCGTTCC | Dm U6 probe 59 nt |
| LL172 | 5'_CGCGAACGATCGGCGAGCGGGGGTGTCATCATTGGTGTCAGCAGCAATGTCCTCACTAG | Dm U4atac probe 59 nt |
| LL173 | 5'_CTAGCCGACCGTTTATGTGTTCCATCCTTGTCTAGGGGAGTGCTAACTTGCTCTCCTTC | Dm U6atac probe 59 nt |
| LL174 | 5'_ggccGTTTCGTGATCGGAAACGt | DmU11 5' ASO F (Not I Xba I pcDNA3.0-MS2) |
| LL175 | 5'_ctagaCGTTTCCGATCACGAAAC | DmU11 5' ASO R (Not I Xba I pcDNA3.0-MS2) |
|  |  |  |
| LL176 | 5'_ttcgCTCTAAAGCTTTCTCACGCA | dU12 cas9 target1 F |
| LL177 | 5'_aaacTGCGTGAGAAAGCTTTAGAG | dU12 cas9 target1 R |
| LL178 | 5'_ttcgGCGTCGCTATGATTCCCAAC | dU12 cas9 target2 F |
| LL179 | 5'_aaacGTTGGGAATCATAGCGACGC | dU12 cas9 target2 R |
| LL180 | 5'_TTCGTGTCGTCAATGTCACGTTGC | DmU6atac cas9 target1 F |
| LL181 | 5'_AAACGCAACGTGACATTGACGACA | DmU6atac cas9 target1 R |
| LL182 | 5'_ttcgGAACACATAAACGGTCGGCT | dU6atac cas9 target2 F |
| LL183 | 5'_aaacAGCCGACCGTTTATGTGTTC | dU6atac cas9 target2 R |
| LL184 | 5'_TTCGaaacgtgatacttgcctgat | DmSmn cas9 target1 F |
| LL185 | 5'_AAACatcaggcaagtatcacgttt | DmSmn cas9 target1 R |
| LL186 | 5'_TTCGaaagaagccagcaccactag | DmSmn cas9 target2 F |
| LL187 | 5'_AAACctagtggtgctggcttcttt | DmSmn cas9 target2 R |
| LL188 | 5'_TTCGGAAACGGATGCCCTGCTCGC | Dm65K N-ter cas9 target1 F |
| LL189 | 5'_AAACGCGAGCAGGGCATCCGTTTC | Dm65K N-ter cas9 target1 R |
| LL190 | 5'_TTCGCCGTAGGTGTTCAGTGAGCG | Dm65K N-ter cas9 target2 F |
| LL191 | 5'_AAACCGCTCACTGAACACCTACGG | Dm65K N-ter cas9 target2 R |
