# Supplementary material for: Defective minor spliceosomes induce SMA-associated phenotypes through sensitive intron-containing neural genes in Drosophila
Source: Nat Commun. 2020 Nov 5;11:5608. doi: 10.1038/s41467-020-19451-z (PMC7644725; doi:10.1038/s41467-020-19451-z)
Supplement: Supplementary file 8 — Reporting Summary [file 41467_2020_19451_MOESM8_ESM.pdf]

nature research

Corresponding author(s): Yong-Zhen Xu, Ph.D.
Wuhan University
Wuhan, Hubei Province CHINA

Last updated by author(s): Sep 5, 2020

Reporting Summary

Nature Research wishes to improve the reproducibility of the work that we publish. This form provides structure for consistency and transparency in reporting. For further information on Nature Research policies, see Authors & Referees and the Editorial Policy Checklist .

Statistics

For all statistical analyses, confirm that the following items are present in the figure legend, table legend, main text, or Methods section.

n/a	Confirmed	
☐	☒	The exact sample size (*n*) for each experimental group/condition, given as a discrete number and unit of measurement
☐	☒	A statement on whether measurements were taken from distinct samples or whether the same sample was measured repeatedly
☐	☒	The statistical test(s) used AND whether they are one- or two-sided<br>*Only common tests should be described solely by name; describe more complex techniques in the Methods section.*
☒	☐	A description of all covariates tested
☒	☐	A description of any assumptions or corrections, such as tests of normality and adjustment for multiple comparisons
☐	☒	A full description of the statistical parameters including central tendency (e.g. means) or other basic estimates (e.g. regression coefficient) AND variation (e.g. standard deviation) or associated estimates of uncertainty (e.g. confidence intervals)
☐	☒	For null hypothesis testing, the test statistic (e.g. *F*, *t*, *r*) with confidence intervals, effect sizes, degrees of freedom and *P* value noted<br>*Give P values as exact values whenever suitable.*
☒	☐	For Bayesian analysis, information on the choice of priors and Markov chain Monte Carlo settings
☒	☐	For hierarchical and complex designs, identification of the appropriate level for tests and full reporting of outcomes
☒	☐	Estimates of effect sizes (e.g. Cohen's *d*, Pearson's *r*), indicating how they were calculated

Our web collection on statistics for biologists contains articles on many of the points above.

Software and code

Policy information about availability of computer code

Data collection	tophat2 version 2.0.9<br>rMATS version 3.2.5<br>RNAduplex version 2.4.3
Data analysis	Clean reads mapped to the Drosophila melanogaster genome (dm6) by TopHat; changes of alternative splicing were analyzed by rMATS. Sequences of 25 nts (positions from -5 to +20) at the 5'SS regions and 20 nts (position from 1 to 20) of the U11 snRNA were selected for measuring stabilities (free energies) of the 5'SS:U11 RNA duplexes using RNAduplex (version 2.4.3).

For manuscripts utilizing custom algorithms or software that are central to the research but not yet described in published literature, software must be made available to editors/reviewers. We strongly encourage code deposition in a community repository (e.g. GitHub). See the Nature Research guidelines for submitting code & software for further information.

Data

Policy information about availability of data

All manuscripts must include a data availability statement. This statement should provide the following information, where applicable:

- Accession codes, unique identifiers, or web links for publicly available datasets
- A list of figures that have associated raw data
- A description of any restrictions on data availability

All raw datasets for transcriptome-wide RNA sequencing have been deposited in NCBI. The accession number is GSE138183.
The data that support the findings of this study are available from the corresponding author upon reasonable request.

Field-specific reporting

Please select the one below that is the best fit for your research. If you are not sure, read the appropriate sections before making your selection.

- [x] Life sciences
- [ ] Behavioural & social sciences
- [ ] Ecological, evolutionary & environmental sciences

For a reference copy of the document with all sections, see nature.com/documents/nr-reporting-summary-flat.pdf

Life sciences study design

All studies must disclose on these points even when the disclosure is negative.

Sample size	For RNA-seq, ~60 M paired-end reads (150 bp) from each sample were obtained and mapped to the Drosophila genome (Table S1).
Data exclusions	No data were excluded.
Replication	For RNA-seq, two biological repeats were tested for each sample; for muscle size and NMJ assays, three larvae from each sample were tested; for locomotion assay, eight larvae were tested.
Randomization	For RNA-seq, each sample contains total RNA from 12 larvae that were randomly picked from vials (~30 homozygous mutant larvae, ~200 wt larvae); for muscle size and NMJ assays, three larvae for each sample were randomly picked from vials (~20 homozygous mutant larvae, ~200 wt larvae); for locomotion assay, eight larvae for each sample were randomly picked from vials (~20 homozygous mutant larvae, ~200 wt larvae).
Blinding	Not applicable.

Reporting for specific materials, systems and methods

We require information from authors about some types of materials, experimental systems and methods used in many studies. Here, indicate whether each material, system or method listed is relevant to your study. If you are not sure if a list item applies to your research, read the appropriate section before selecting a response.

Materials & experimental systems

n/a	Involved in the study	
[ ]	[x]	Antibodies
[ ]	[x]	Eukaryotic cell lines
[x]	[ ]	Palaeontology
[ ]	[x]	Animals and other organisms
[x]	[ ]	Human research participants
[x]	[ ]	Clinical data

Methods

n/a	Involved in the study	
[x]	[ ]	ChIP-seq
[x]	[ ]	Flow cytometry
[x]	[ ]	MRI-based neuroimaging

Antibodies

Antibodies used	4F3 anti-discs large (DSHB); Alexa Fluor 647 conjugated Rabbit Anti-Mouse IgG (Jackson Immuno Research); Alexa Fluor 488 conjugated goat anti-HRP IgG (Jackson Immuno Research); Monoclonal ANTI-FLAG M2-Peroxidase (HRP) antibody (Sigma); Anti-α-Tubulin antibody (Sigma); Anti-SNRPD2 (Abcam); Monoclonal Anti-Rabbit IgG (Sigma); Anti-Mouse IgG (Sigma).
Validation	All primary antibodies were validated by western blot.

Eukaryotic cell lines

Policy information about cell lines

Cell line source(s)	Drosophila S2 cell
Authentication	Splicing of sex determination pathway genes, including sxl, tra, and dsx, were tested, and showed male-specific pattern. This is consistent with the male specificity of Drosophila S2 cell.
Mycoplasma contamination	Not tested.
Commonly misidentified lines (See ICLAC register)	Not applicable.

Animals and other organisms

Policy information about studies involving animals; ARRIVE guidelines recommended for reporting animal research

Laboratory animals	flies:<br>w1118<br>w1118/Dp(1;Y)y+; TM2/TM6C,Sb1<br>CyO/snaSco<br>dgo308/CyO, P{GFP-un1}CyO<br>Sb1/TM3, P{GAL4-Hsp70. PB}, P{UASGFP.Y}, Ser1<br>otu7/FM7c<br>attp40{UAS-SmnshRNA}<br>SmnΔ/ CyO<br>U12Δ/ TM6C, Sb1<br>U6atacΔ/ CyO<br>P{GAL4-da.G32}UH1/TM3, Sb1<br>P{GawB}elavC155<br>FTH-Dm65K<br>P{GawB}elavC155; SmnΔ/ TM3, P{GAL4-Hsp70.PB}, P{UAS-GFP.Y}, Ser1<br>UAS-Smn; SmnΔ/TM3, P{GAL4-Hsp70.PB}, P{UAS-GFP.Y}, Ser1<br>UAS-ZMYND10; SmnΔ/ TM3, P{GAL4-Hsp70.PB}, P{UAS-GFP.Y}, Ser1<br>UAS-CG10171; SmnΔ/TM3, P{GAL4-Hsp70.PB}, P{UAS-GFP.Y}, Ser1<br>UAS-Pcyt2; SmnΔ/ TM3, P{GAL4-Hsp70.PB}, P{UAS-GFP.Y}, Ser1<br>UAS-Fas3; SmnΔ/TM3, P{GAL4-Hsp70.PB}, P{UAS-GFP.Y}, Ser1<br>UAS-CG16984; SmnΔ/TM3, P{GAL4-Hsp70.PB}, P{UAS-GFP.Y}, Ser1<br>UAS-galectin; SmnΔ/TM3, P{GAL4-Hsp70.PB}, P{UAS-GFP.Y}, Ser1<br>UAS-MCPH1; SmnΔ/TM3, P{GAL4-Hsp70.PB}, P{UAS-GFP.Y}, Ser1<br>UAS-Plc21C; SmnΔ/TM3, P{GAL4-Hsp70.PB}, P{UAS-GFP.Y}, Ser1<br>UAS-sgg; SmnΔ/TM3, P{GAL4-Hsp70.PB}, P{UAS-GFP.Y}, Ser1<br>UAS-Fer2; SmnΔ/TM3, P{GAL4-Hsp70.PB}, P{UAS-GFP.Y}, Ser1<br>UAS-Got2; SmnΔ/TM3, P{GAL4-Hsp70.PB}, P{UAS-GFP.Y}, Ser1<br>UAS-rut; SmnΔ/TM3, P{GAL4-Hsp70.PB}, P{UAS-GFP.Y}, Ser1<br>UAS-Pde1c; SmnΔ/TM3, P{GAL4-Hsp70.PB}, P{UAS-GFP.Y}, Ser1<br>UAS-sei; SmnΔ/TM3, P{GAL4-Hsp70.PB}, P{UAS-GFP.Y}, Ser1<br>UAS-Pdk1; SmnΔ/TM3, P{GAL4-Hsp70.PB}, P{UAS-GFP.Y}, Ser1<br>UAS-Pka-R2; SmnΔ/TM3, P{GAL4-Hsp70.PB}, P{UAS-GFP.Y}, Ser1<br>UAS-cnn; SmnΔ/TM3, P{GAL4-Hsp70.PB}, P{UAS-GFP.Y}, Ser1<br>UAS-Cnb; SmnΔ/TM3, P{GAL4-Hsp70.PB}, P{UAS-GFP.Y}, Ser1<br>UAS-KaiR1D; SmnΔ/TM3, P{GAL4-Hsp70.PB}, P{UAS-GFP.Y}, Ser1<br>w1118; Df(2L)ED629, P{3'.RS5+3.3'}ED629/SM6a<br>w1118; Df(3L)BSC561/TM6C, Sb1
Wild animals	Not applicable.
Field-collected samples	Not applicable.
Ethics oversight	Not applicable.

Note that full information on the approval of the study protocol must also be provided in the manuscript.
